# BMP4 activates the Wnt–*Lin28A*–*Blimp1*–Wnt pathway to promote primordial germ cell formation via altering H3K4me2

**DOI:** 10.1242/jcs.249375

**Published:** 2021-02-01

**Authors:** Qisheng Zuo, Kai Jin, Man Wang, Yani Zhang, Guohong Chen, Bichun Li

**Affiliations:** 1 Key Laboratory of Animal Breeding Reproduction and Molecular Design for Jiangsu Province, College of Animal Science and Technology, Yangzhou University, Yangzhou, Jiangsu 225009, China; 2 Joint International Research Laboratory of Agriculture and Agri-Product Safety, The Ministry of Education of China, Yangzhou University, Yangzhou, Jiangsu 225009, China

**Keywords:** Chicken, Primordial germ cells, H3K4me2, *Lin28A*, Wnt signaling

## Abstract

The unique developmental characteristics of chicken primordial germ cells (PGCs) enable them to be used in recovery of endangered bird species, gene editing and the generation of transgenic birds, but the limited number of PGCs greatly limits their application. Studies have shown that the formation of mammalian PGCs is induced by BMP4 signal, but the mechanism underlying chicken PGC formation has not been determined. Here, we confirmed that Wnt signaling activated via BMP4 activates transcription of *Lin28A* by inducing β-catenin to compete with LSD1 for binding to TCF7L2, causing LSD1 to dissociate from the *Lin28A* promoter and enhancing H3K4me2 methylation in this region. *Lin28A* promotes PGC formation by inhibiting *gga-let7a-3p* maturation to initiate *Blimp1* expression. Interestingly, expression of *Blimp1* helped sustain *Wnt5A* expression by preventing LSD1 binding to the *Wnt5A* promoter. We thus elucidated a positive feedback pathway involving Wnt–*Lin28A*–*Blimp1*–Wnt that ensures PGC formation. In summary, our data provide new insight into the development of PGCs in chickens*.*

## Abstract (precis)

**Summary:** We describe a Wnt–*Lin28A*–*Blimp1*–Wnt positive feedback regulation system during the formation of primordial germ cells, which is regulated by upstream BMP4 signals and H3K4me2.

## INTRODUCTION

Primordial germ cells (PGCs) are the progenitors of sperm and ovum, which are responsible for the transmission of genetic information between germ lines (Liu et al., 2018; Nakamura, 2017). Chicken PGCs have the characteristic of migrating to the genital ridge via the blood, which enables their wide application in recovery of endangered bird species, gene editing and the generation of transgenic birds (Lu et al., 2014; Ono and Machida, 1999; Zhao and Kuwana, 2003). Therefore, studying the molecular mechanisms underlying chicken PGC formation could facilitate the application of PGCs. The molecular mechanisms underlying PGC formation in mammals have been well studied; however, such research in chicken PGCs has been limited. Additionally, because of species differences, it is not clear whether the mechanisms of PGC formation in mammals can be applied to chickens.

Two modes for the origin of PGCs have been described: epigenesis and preformation (Lasko and Ashburner, 1988; Wentworth et al., 1989). The formation of PGCs in mammals occurs via epigenesis. During the embryonic day (E)5.5–E6.25 stage of mouse embryo development, some of the mesoderm cells induced by the BMP4 signal secreted by the ectoderm begin to express genes such as *Blimp1* (also known as *Prdm1*) and *Tcfap2c*, differentiate into PGCs, then migrate from the bottom of the allantoic sac to the endoderm and finally settle in the genital ridge (Saitou and Yamaji, 2012; Yamaguchi et al., 2000). The formation of PGCs in invertebrates such as *Drosophila* occurs via preformation. The origin of PGCs in *Drosophila* is influenced by germplasm RNA (maternal genetic factor). Within 1.5 h to 3 h of laying eggs, approximately ten PGC precursors already exist in the embryo tail of *Drosophila*, which is completely different from that of mammals (Spudich et al., 1998). Chicken PGCs originate from the ectoderm, then gradually transfer to the endoderm and finally migrate to the genital ridge [Hamburger and Hamilton (HH) stage 10–12] through the blood (Aige-Gil and Simkiss, 1991; Vick et al., 1993). The formation of PGCs in chickens is different from that in *Drosophila* and mammals, but which mode it occurs via needs further exploration.

At present, the molecular mechanisms underlying PGC formation in mice are relatively clear. Studies (Lawson et al., 1999; Saitou et al., 2002) reported induction of PGC [*Fraglis^+^* (also known as *Ifitm3*^+^) *Blimp1^+^*] formation from mouse precursor cells in the proximal epidermis mediated via BMP4 signaling. Inhibition of BMP4 signaling markedly decreases the number of PGCs in mouse gonads (De Felici, 2016; Miyauchi et al., 2017; Ying et al., 2001). However, other studies (Aramaki et al., 2013; Bialecka et al., 2012; Cervantes et al., 2009) confirmed that Wnt signaling also plays an important role in PGC formation in mice. BMP4 and Wnt signaling interact during PGC formation by activating *Blimp1* via the mesoderm-specific transcription factor T (Aramaki et al., 2013; Chassot et al., 2017; Yamaguchi et al., 1999). Specifically, in mouse epidermis, activation of BMP4 signaling and suppression of Wnt signaling leads to partial or complete loss of marker gene (such as *Blimp1*) expression in early PGCs (Ohinata et al., 2009; Saitou et al., 2005), whereas activation of Wnt signaling enhances the epidermal response to BMP4 signaling. However, until now, how BMP4 and Wnt signaling activate *Blimp1* expression to regulate PGC formation and how these signaling pathways interact remain to be elucidated.

In a previous study, we found that BMP4 and Wnt signaling are significantly upregulated during chicken PGC formation via RNA sequencing (RNA-seq) of chicken embryonic stem cells (ESCs) and PGCs (Zhang et al., 2015). Functional verification analyses demonstrated that BMP4 signaling promotes the formation of chicken PGCs (Zuo et al., 2017). However, the role of Wnt signaling in PGC formation in chickens and whether Wnt and BMP4 interact have yet to be determined. Here, we demonstrate that Wnt signaling positively regulates PGC formation. Our results also indicate that PGC formation is controlled by a Wnt–*Lin28A*–*Blimp1*–Wnt positive-feedback loop regulated by upstream BMP4 signaling and H3K4me2. Our results lay the foundation for the systematic analysis of the molecular mechanisms underlying chicken PGC formation, and also provide a new perspective to elucidate the origin of chicken PGCs.

## RESULTS

### Wnt5A–β-catenin–TCF7L2 positively regulate the formation of PGCs

**Fig. 1.**
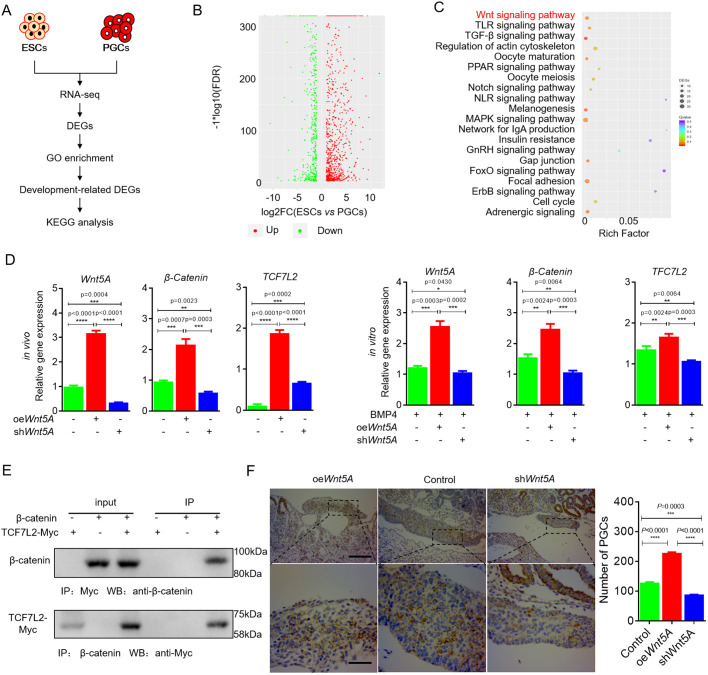
**Wnt5A–β-catenin–TCF7L2 positively regulates the formation of PGCs.** (A) Schematic diagram of RNA-seq analysis of embryonic stem cells (ESCs) and primordial germ cells (PGCs). DEG, differentially expressed genes; GO, gene ontology; KEGG, Kyoto Encyclopedia of Genes and Genomes. (B) Volcano plot of DEGs of ESCs and PGCs. FDR, false discovery rate. (C) KEGG analysis of DEGs in ESCs and PGCs. (D) Detection of β-catenin and *Tcf7l2* expression by qRT-PCR after *Wnt5A* overexpression and interference *in vivo* (left) and *in vitro* (right). (E) Co-IP results show that β-catenin and TCF7L2 can bind to each other. (F) IHC in the genital ridge by anti-CVH antibody. Scale bars: 200 μm (top row), 40 μm (bottom row). *P<0.05, **P<0.01, ***P<0.001, ****P<0.0001 (two-sample paired Student's *t*-tests).

In order to analyze the molecular mechanism regulating chicken PGC formation, the differentially expressed genes (DEGs) of ESCs and PGCs were identified by RNA-seq (Fig. 1A,B). Gene ontology (GO) analysis showed that 2516 DEGs were enriched in development-related terms (Fig. S1A,B and Table S1), 20 genes of which were significantly enriched in the Wnt signaling pathway (*P*=0.0023) (Fig. 1C; Table S2). Results from quantitative reverse transcription PCR (qRT-PCR) also confirmed that the key molecules of Wnt signaling were significantly activated in the process of chicken PGC formation (Fig. S1C,D) (Zuo et al., 2018b). These results indicate the involvement of Wnt signaling in PGC formation.

We determined that *Wnt5A*, β-catenin and *Tcf7l2* are the key Wnt signaling molecules in the formation of chicken germ stem cells (Fig. S1C,D) (He et al., 2018). To further demonstrate the involvement of Wnt5A–β-catenin–TCF7L2 signaling, we conducted *Wnt5A* overexpression/interference during the process of inducing ESCs to form PGCs with BMP4. The expression of signaling molecules in 4 day-induced cells was assessed by qRT-PCR, which indicated significant downregulation of β-catenin and *Tcf7l2* expression (*P*<0.01) following *Wnt5A* interference, and significant upregulation of β-catenin and *Tcf7l2* expression (*P*<0.01) following *Wnt5A* overexpression (Fig. 1D). *In vivo* experiments produced similar results (Fig. 1D). Co-immunoprecipitation (Co-IP) of lysates of PGCs co-transfected with pcDNA3.1-Myc-*Tcf7l2* and pcDNA3.1-β-catenin revealed that TCF7L2 and β-catenin can combine with each other (Fig. 1E). These data indicated that Wnt5A mediates Wnt5A–β-catenin–TCF7L2 signaling in PGCs. Then, we confirmed that overexpressing *Wnt5A* promotes PGC formation; however, contrasting results were obtained after interference with *Wnt5A* (Fig. 1F). Thus, we come to the conclusion that Wnt5A–β-catenin–TCF7L2 positively regulates PGC formation.

### *Lin28A* is a specific target of Wnt5A signaling in PGCs

**Fig. 2.**
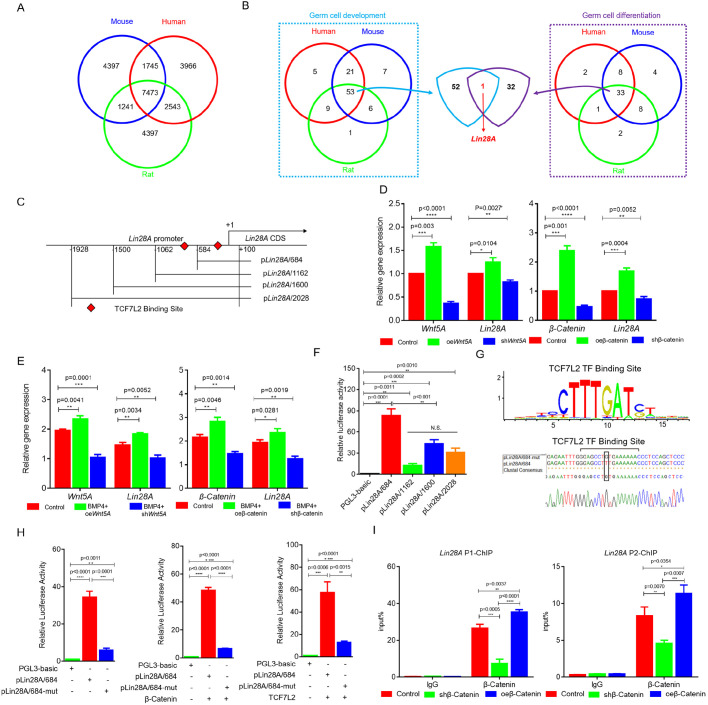
***Lin28A* is a specific target gene of Wnt5A signal in PGCs.** (A) Venn diagram analysis of TCF7L2 target genes of human (15,727), rat (15,652) and mouse (15,374) enriched from GTRD. (B) GO analysis was performed on 7473 target genes, and *Lin28A* was screened from GO entries of germ cell development and germ cell differentiation. (C) Vector construction of different length fragments of the *Lin28A* promoter. Red diamonds indicate the TCF7L2 binding site. (D,E) The expression of *Lin28A* was detected by qRT-PCR after *Wnt5A* and β-catenin were overexpressed or interfered with during the formation of PGCs *in vitro* (D) and *in vivo* (E). (F) Screening of core regions of the *Lin28A* promoter by the dual luciferase system. (G) Mutation of the TCF7L2 binding site in the *Lin28A* promoter region. Top: TCF7L2 binding site consensus motif. Middle: part of the *Lin28* promoter region, showing the mutation introduced (box). Bottom: representative sequencing result. (H) The effect of TCF7L2 mutation on activity of the *Lin28A* promoter was detected by the dual luciferase system in DF-1 cells. (I) ChIP-qPCR was used to detect the enrichment of TCF7L2 in the *Lin28A* promoter region. *P<0.05; **P<0.01; ***P<0.001; ****P<0.0001; N.S., not significant (two-sample paired Student's *t*-tests).

To identify the specific target genes regulated by Wnt during PGC formation, we examined the enrichment of target genes of TCF7L2 in humans (15,727), rat (15,652) and mouse (15,374) in the Gene Transcription Regulation Database (GTRD) (no bird database available) (Fig. 2A). *Lin28A* was the only common gene in GO and Venn diagram analyses of 7473 target genes identified in these species (Fig. 2B; Fig. S2), suggesting that *Lin28A*, a highly conserved gene targeted by Wnt signal, is involved in the generation of reproductive stem cells. These results are consistent with previous reports in mammals (Bazley et al., 2015; Matzuk, 2009). Conservative analysis of the gene structure showed that chicken and mammalian *Lin28A* were exactly the same (Fig. S3), and the binding site for TCF7L2 was also presented in the chicken *Lin28A* promoter (Fig. 2C). Such results indicate that *Lin28A* is a target of Wnt signal, which may be involved in the formation of chicken PGCs.

To confirm that *Lin28A* was regulated by Wnt signaling, we examined the expression of *Lin28A* after activation/inhibition of Wnt signaling during PGC formation *in vivo*. Overexpression of *Wnt5A* significantly increased *Lin28A* expression, whereas inhibition of *Wnt5A* expression significantly inhibited *Lin28A* expression (Fig. 2D). *Lin28A* expression also decreased following inhibition of β-catenin, and increased following overexpression of β-catenin (Fig. 2D). Similar results were observed in induction experiments *in vitro* (Fig. 2E). Collectively, these results indicate that *Lin28A* responds to Wnt signaling. To examine this response further, we identified the core promoter of *Lin28A* (−584 to +100 bp) using the dual luciferase detection system (Fig. 2C,F). Activation of Wnt signaling (overexpression of β-catenin) significantly increased the activity of the *Lin28A* promoter (*P*<0.01) (Fig. 2F). However, mutation of the TCF7L2 binding site significantly reduced the activity of the *Lin28A* promoter (*P*<0.01) (Fig. 2G,H). Activation of Wnt signaling could not rescue *Lin28A* promoter activity following introduction of point mutations (Fig. 2H), indicating that *Lin28A* responds to Wnt signaling via the TCF7L2 binding site in the promoter. Subsequently, we examined the binding of TCF7L2 to the *Lin28A* promoter using chromatin immunoprecipitation (ChIP) coupled with quantitative PCR (ChIP-qPCR) and found enrichment of β-catenin–TCF7L2 complexes in the *Lin28A* promoter (Fig. 2I). Activation of Wnt signaling significantly increased binding of the β-catenin–TCF7L2 complex to *Lin28A* (*P*<0.01), whereas inhibition of Wnt signaling significantly reduced this binding (*P*<0.01) (Fig. 2I). These results indicate that *Lin28A* is a downstream target of Wnt signaling.

### *Lin28A* positively regulates PGC formation *in vitro* and *in vivo*

**Fig. 3.**
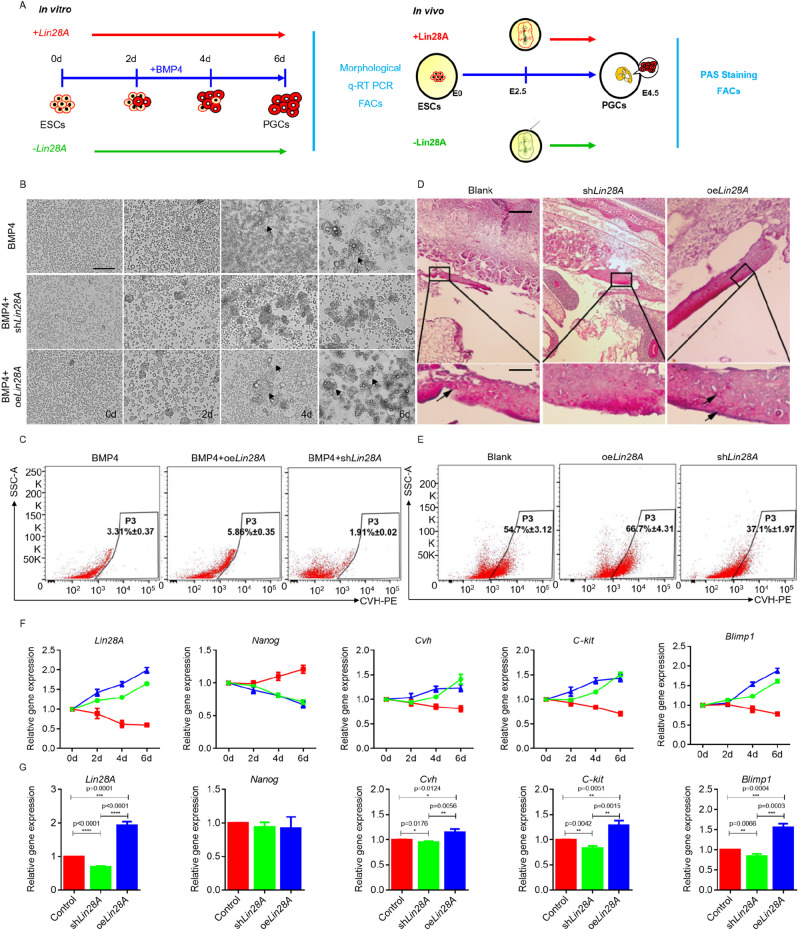
***Lin28A* positively regulates PGC formation *in vitro* and *in vivo*.** (A) Schematic diagrams of functional verification of Lin28A in the formation of PGCs *in vitro* (left) and *in vivo* (right). FACS, fluorescence-activated cell sorting; PAS, periodic acid–Schiff. (B) Morphological observation of the number of embryoid bodies in the PGC induction model *in vitro* after *Lin28A* overexpression and interference. Scale bar: 60 μm. (C,E) Detection of PGC formation efficiency after *Lin28A* overexpression or interference *in vitro* (C) and *in vivo* (E) by flow cytometry. (D) Number of PGCs in the genital ridge after *Lin28A* overexpression or interference *in vitro* were detected by PAS staining. Scale bars: 200 μm (top row), 40 μm (bottom row). (F,G) The expression of reproductive marker genes was detected by qRT-PCR after *Lin28A* overexpression and interference *in vitro* (F) and *in vivo* (G).

Next, we investigated the function of *Lin28A* in PGC formation (Fig. 3A). *Lin28A* was inhibited or overexpressed during BMP4-induced differentiation of ESCs into PGCs *in vitro* (Fig. 3A; Fig. S4A). Morphologic observations on day 2 after BMP4 induction indicated that the cells had begun to grow larger. A few embryoid bodies (EBs) appeared on day 4, and the number of EBs increased on day 6; however, no EBs appeared between days 2 and 6 after *Lin28A* inhibition. In contrast, small EBs began to appear on day 2 after *Lin28A* overexpression, and on day 4 these EBs became larger and began to break. The number of EBs increased on day 6, the cell edges began to rupture, and a few cells were released from the EBs (Fig. 3B; Fig. S4B). *Lin28A* overexpression significantly decreased expression of the pluripotency marker gene *Nanog* and increased *Cvh*, *C-kit* and *Blimp1* expression. Flow cytometry analyses demonstrated that *Lin28A* overexpression promoted PGC formation in the BMP4 model (Fig. 3C,F,G). Similar results were observed in *in vivo* experiments (Fig. 3D,E; Fig. S4C). Periodic acid–Schiff staining (PAS) was used to monitor changes in the number of PGCs formed in the genital ridge after *Lin28A* overexpression/interference. Compared with the number of PGCs in the genital ridge during the normal *in vivo* hatching process (38±1.53), the number of PGCs in the genital ridge significantly increased following *Lin28A* overexpression (46±2.10; *P*<0.01) and significantly decreased (20±1.64; *P*<0.01) following *Lin28A* interference (Fig. 3D; Fig. S4C). Collectively, these results indicate that Wnt–β-catenin signaling promotes PGC formation by activating *Lin28A* expression.

### *Lin28A* is regulated by H3K4me2

**Fig. 4.**
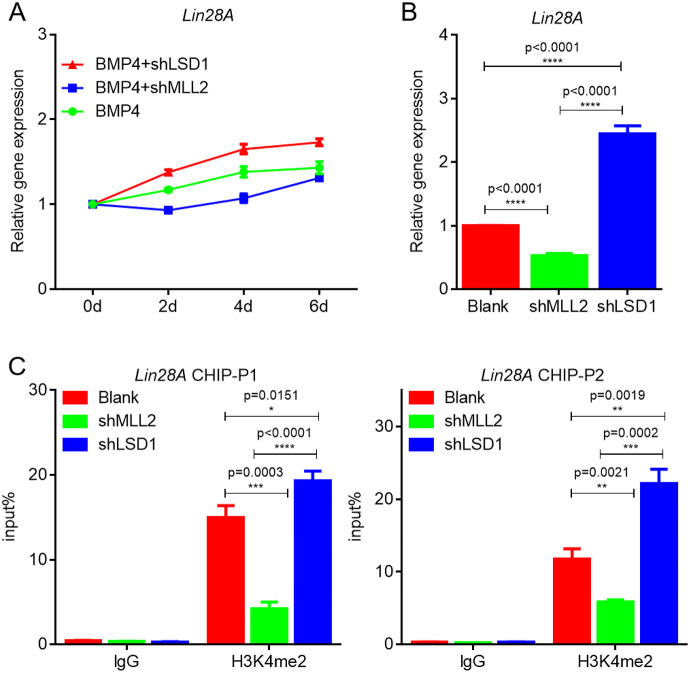
***Lin28A* is a target gene regulated by H3K4me2. (**A) Detection of *Lin28A* expression after interference with *Lsd1* and *Mll2* in the BMP4 induction model. (B) Detection of *Lin28A* expression after interference with *Lsd1* and *Mll2* in PGCs. (C) ChIP-qPCR was used to detect the enrichment level of H3K4me2 in the *Lin28A* promoter region after interference with *Lsd1* and *Mll2* in PGCs. P1 and P2 present different loci of H3K4me2 in the *Lin28A* promoter region. *P<0.05, **P<0.01, ***P<0.001, ****P<0.0001 (two-sample paired Student's *t*-tests).

**Fig. 5.**
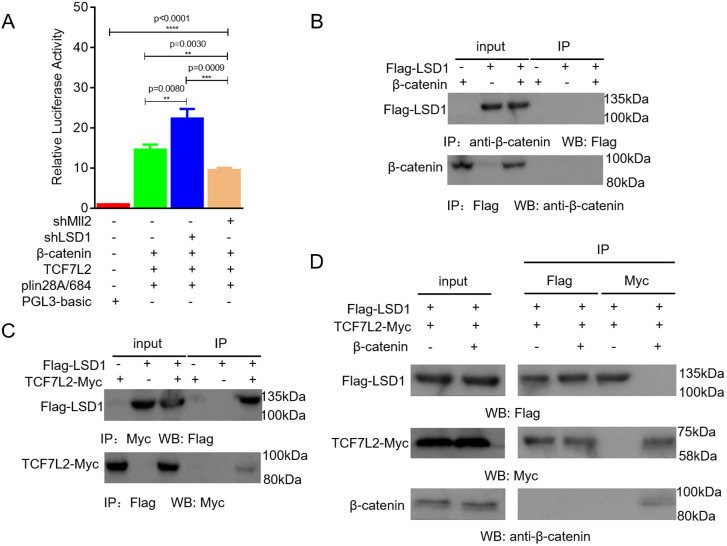
**Competition between β-catenin and LSD1 for TCF7L2 regulates the expression of *Lin28A* during the formation of PGCs.** (A) Effects of Wnt signaling on the regulation of *Lin28A* promoter activity after *Mll2* and *Lsd1* interference were detected by the dual luciferase system. (B,C) Results from Co-IP showed that LSD1 cannot bind to β-catenin (B), but binds to TCF7L2 (C), in PGCs. (D) Results from Co-IP showed that β-catenin competes with LSD1 for binding to TCF7L2 in PGCs. **P<0.01, ***P<0.001, ****P<0.0001 (two-sample paired Student's *t*-tests).

In a separate study on H3K4me2 regulating spermatogenic stem cell (SSC) formation (data not shown), we performed RNA-seq on SSCs treated with LSD1 (H3K4me2 demethylation-modifying enzyme; see Data availability section). It was found that *Lin28A* was one of the DEGs (Fig. S5A). Furthermore, results from qRT-PCR showed that expression of *Lin28A* was significantly upregulated after interference with *Lsd1* in SSCs (Fig. S5B). ChIP-qPCR results also showed that the H3K4me2 enrichment level in the *Lin28A* promoter region was regulated by LSD1 (Fig. S5C). These results suggest that *Lin28A* may be the target gene of H3K4me2. To examine the effect of H3K4me2 on *Lin28A* regulation during PGC formation in the present study, we interfered with *Lsd1* and *Mll2* expression in the BMP4 induction model *in vitro* (Zhang et al., 2020)*. Lin28A* expression was significantly higher than that induced by BMP4 after interfering with *Lsd1* expression, and the opposite trend was observed after interfering with *Mll2* (Fig. 4A), indicating that H3K4me2 positively regulates *Lin28A* transcription *in vitro*. The experiments *in vivo* provided similar results (Fig. 4B), suggesting that *Lin28A* is also regulated by H3K4me2 in PGCs. To confirm that *Lin28A* is a target of H3K4me2, we examined the level of H3K4me2 enrichment in the *Lin28A* promoter in PGCs using ChIP-qPCR. Compared with the control, H3K4me2 in the *Lin28A* promoter was significantly downregulated following *Mll2* interference and significantly upregulated following *Lsd1* interference (Fig. 4C). Further results confirmed that changes in H3K4me2 regulate the activity of the *Lin28A* promoter (Fig. 5A). Collectively, these results indicate that, in addition to Wnt signaling, H3K4me2 also regulates *Lin28A* expression during PGC formation.

### Competition between β-catenin and LSD1 for TCF7L2 regulates *Lin28A* expression during PGC formation

To further elucidate the molecular mechanism regulating *Lin28A* expression, we investigated the interaction between the Wnt signal and H3K4me2 in regulating the expression of *Lin28A* by the dual luciferase system. Interference with *Mll2* expression suppressed the response of *Lin28A* to Wnt signaling, whereas interference with *Lsd1* significantly enhanced the response (Fig. 5A). The position of H3K4me2 enrichment in the *Lin28A* promoter is near the TCF7L2 binding site. It is reasonable to speculate that β-catenin–TCF7L2 complexes affect the level of H3K4me2 enrichment to regulate *Lin28A* expression by altering the binding of LSD1 or MLL2 to the *Lin28A* promoter. Considering that the complex involving MLL2 is relatively fixed (Glaser et al., 2009; Steward et al., 2006), we used Co-IP to assess the interactions between β-catenin, TCF7L2 and LSD1. Co-IP performed after co-transfection of DF1 cells and PGCs with LSD1-Flag and β-catenin vectors indicated no interaction between LSD1 and β-catenin (Fig. 5B; Fig. S6A). However, in cells co-transfected with LSD1-Flag and TCF7L2-Myc, interaction between Flag and TCF7L2 was observed (Fig. 5C; Fig. S6B). Considering the correlation between TCF7L2 and β-catenin (Hou et al., 2016), we hypothesized that, in ESCs, TCF7L2 binding in the *Lin28A* promoter recruits LSD1, which reduces the level of H3K4me2 enrichment, inhibiting *Lin28A* transcription; during PGC formation, β-catenin enters the nucleus ectopically and competes with LSD1 for binding to TCF7L2, which increases the level of H3K4me2 enrichment and promotes *Lin28A* transcription. To test this hypothesis, DF1 cells and PGCs were co-transfected with LSD1-Flag, TCF7L2-Myc and β-catenin vectors. Co-IP indicated that LSD1 did not bind to TCF7L2, whereas β-catenin did bind to TCF7L2 (Fig. 5D; Fig. S6C). Collectively, these results indicated that β-catenin competes with LSD1 for binding to TCF7L2, which demethylates H3K4me2 in the *Lin28A* promoter via LSD1 and activates *Lin28A* expression during PGC formation.

### *Lin28A* activates *Blimp1* to regulate PGC formation by inhibiting *gga-let-7a-2-3p* maturation

**Fig. 6.**
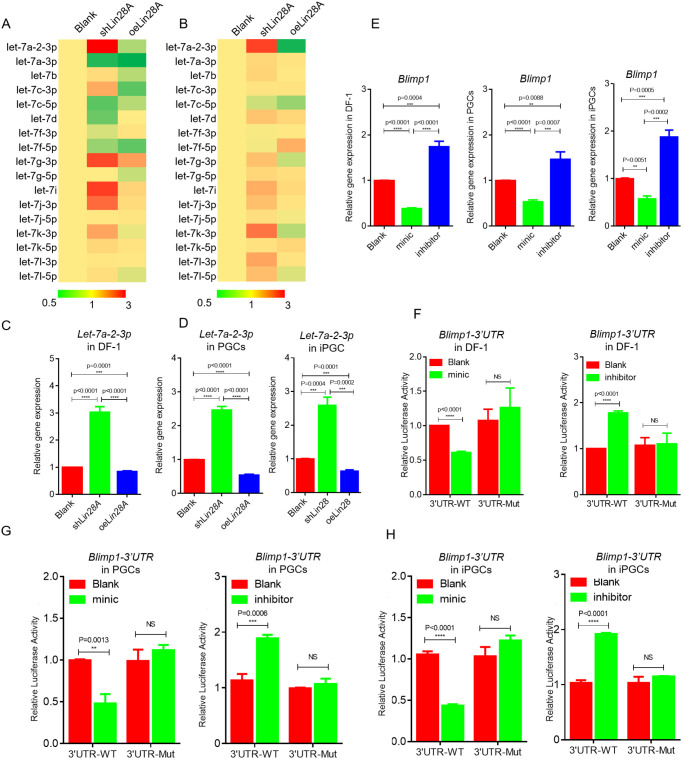
***Lin28A* activates *Blimp1* to regulate the formation of PGCs by inhibiting *gga-let-7a-2-3p* maturation.** (A,B) qRT-PCR was used to detect the expression of *let7* microRNAs after *Lin28A* overexpression and interference in ESCs (A) and PGCs (B). (C,D) qRT-PCR was used to detect the expression of *Let-7a-2-3p after Lin28A* overexpression and interference in DF-1 cells (C), PGCs (D, left) and iPGCs (D, right). (E) qRT-PCR was used to detect the expression of *Blimp1* after mimic and inhibitor of *gga-let-7a-2-3p* were transfected into DF-1 cells (left), PGCs (middle) and induced PGCs (iPGCs; right). (F–H) The dual luciferase system was used to detect that *gga-let-7a-2-3p* regulates *Blimp1* 3'UTR activity in DF-1 cells (F), PGCs (G) and iPGCs (H). **P<0.01; ***P<0.001; ****P<0.0001; NS, not significant (two-sample paired Student's *t*-tests).

Matzuk (2009) demonstrated that, as an RNA-binding protein, Lin28A regulates the expression of related genes by inhibiting *let7* microRNA (miRNA) maturation. However, the *let7* miRNA that interacts with *Lin28A* during chicken PGC formation has yet to be identified. To determine the key *let7* miRNAs targeted b*y Lin28A*, 17 *gga-let7* sequences in the chicken *let7* miRNA family were screened using miRDB (Fig. 6A,B). To identify specific *let7* miRNAs interacting with *Lin28A*, the expression of mature *let7* miRNAs in chicken ESCs and PGCs was evaluated by qRT-PCR after *Lin28A* overexpression/interference (Fig. 6A,B). The results indicated that *gga-let-7a-2-3p* was significantly regulated by *Lin28A* in ESCs and PGCs (Fig. 6A,B). *gga-let-7a-2-3p* was significantly upregulated following *Lin28A* overexpression and significantly downregulated following *Lin28A* interference (Fig. 6C,D) in DF-1 cells, PGCs and induced PGCs (iPGCs). Combined with the results from previous studies (Matzuk, 2009), we conclude that *gga-let-7a-2-3p* of *let7* miRNA can interact with *Lin28A* during the formation of PGCs in chickens.

Screening the miRDB identified 1143 genes targeted by *gga-let-7a-2-3p*. In particular, *Blimp1*, which plays an important regulatory role in PGC formation, attracted our attention (Lin et al., 2014; Murakami et al., 2016). To determine whether *gga-let-7a-2-3p* targets *Blimp1*, we synthesized a *gga-let-7a-2-3p* mimic and inhibitor and transfected them into DF1 cells. qRT-PCR analysis indicated that *Blimp1* expression was significantly downregulated in DF1 cells transfected with the mimic (Fig. 6E) and significantly upregulated in cells transfected with the inhibitor, indicating that *gga-let-7a- 2-3p* negatively regulates *Blimp1* (*P*<0.01 for both). As *Blimp1* is a PGC marker, we performed the same experiment with PGCs and iPGCs and obtained similar results (Fig. 6E). To further confirm that *gga-let-7a-2-3p* targets *Blimp1*, we predicted the *gga-let-7a-2-3p* binding site in the *Blimp1* 3'UTR (UUGUACA). Wild-type (WT) and mutant (complete deletion of binding site) luciferase reporter vectors of the *Blimp1* 3'UTR were constructed separately. DF1 cells were then co-transfected with vectors for the *gga-let-7a-2-3p* mimic and inhibitor with *Blimp1*-3'UTR-WT and *Blimp1*-3'UTR-Mut. The *gga-let-7a-2-3p* inhibitor significantly increased *Blimp1*-3'UTR-WT luciferase activity in the double luciferase reporter assay (*P*<0.01), but had no significant effect on *Blimp1*-3'UTR-Mut (*P*>0.05) (Fig. 6F). The *gga-let-7a-2-3p* mimic significantly reduced *Blimp1*-3'UTR-WT luciferase activity (*P*<0.01), but had no significant effect on *Blimp1*-3'UTR-Mut (*P*>0.05) (Fig. 6F). The same regulatory pattern was detected in PGCs and iPGCs (Fig. 6G,H). These results indicated that *Blimp1* is a direct target of *gga-let-7a* and that *gga-let-7a* binds to the 3'UTR of *Blimp1* to inhibit its expression.

### *Blimp1* interacts with LSD1 to regulate the expression of related genes in Wnt signaling and participates in PGC formation

**Fig. 7.**
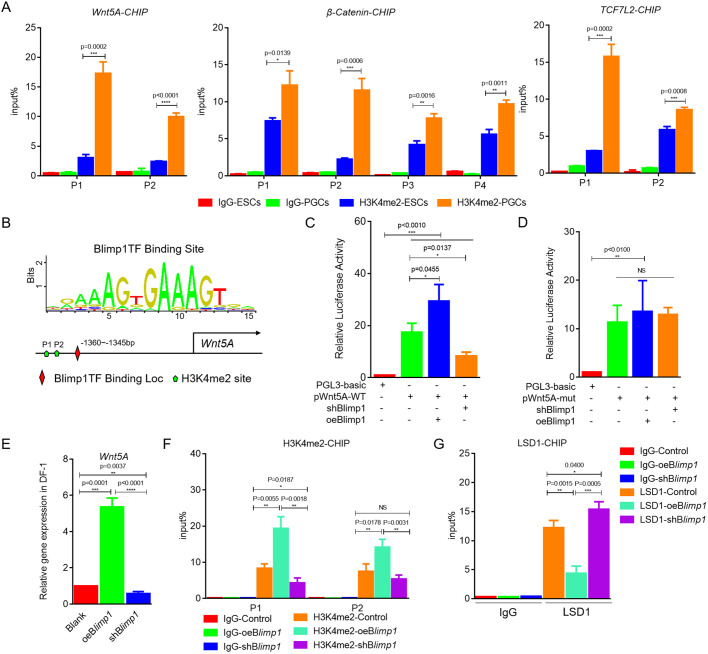
**Blimp1 interacts with LSD1 to regulate the expression of related genes in Wnt signaling and participate in the formation of PGCs.** (A) Detection of H3K4me2 enrichment in *Wnt5A* (left), β-catenin (middle) and *Tcf7l2* (right) promoter regions in ESCs and PGCs by ChIP-qPCR. (B) Schematic diagram of the Blimp1 binding site and H3K4me2 enrichment site in the *Wnt5A* promoter region. (C,D) The effects of *Blimp1* overexpression or interference on activity of wild-type (WT) (C) and mutant (D) *Wnt5A* promoter were detected by the dual luciferase reporter system. (E) Detection of *Wnt5A* expression after *Blimp1* overexpression or interference by qRT-PCR. (F) Detection of H3K4me2 enrichment in *Wnt5A* promoter regions after *Blimp 1* overexpression or interference by ChIP-qPCR. (G) Detection of LSD1 enrichment in *Wnt5A* promoter regions after *Blimp1* overexpression or interference by ChIP-qPCR. *P<0.05; **P<0.01; ***P<0.001; ****P<0.0001; NS, not significant (two-sample paired Student's *t*-tests).

As *Blimp1* is known to affect the level of H3K4me2 (Minnich et al., 2016), we asked whether Blimp1 regulates the formation of PGCs by changing the H3K4me2 level in the promoter region of key genes. Correlation between H3K4me2 and Wnt signaling was examined during PGC formation. Results from ChIP-qPCR showed two, four and two H3K4me2 enrichment sites in the *Wnt5A*, β-catenin and *Tcf7l2* promoters, respectively. PGCs exhibited significantly higher binding of H3K4me2 than ESCs (*P*<0.01) (Fig. 7A), which was regulated by LSD1 and MLL2 (Fig. S7), indicating that H3K4me2 regulates key Wnt signaling molecules. We then investigated whether Blimp1 regulates H3K4me2 in the promoters of *Wnt5A*, β-catenin and *Tcf7l2*. Notably, there is a Blimp1 binding site near the *Wnt5A* promoter H3K4me2 enrichment site (Fig. 7B). To confirm that Blimp1 binds to the *Wnt5A* promoter, a double luciferase reporter vector for the *Wnt5A* promoter was constructed and co-transfected into DF1 cells along with *Blimp1* overexpression/interference vectors. The double luciferase reporter assay showed that *Blimp1* overexpression significantly enhanced *Wnt5A* promoter activity, whereas interference with *Blimp1* expression decreased promoter activity (Fig. 7C). However, *Blimp1* overexpression/interference had no effect on promoter activity after mutation of the Blimp1 binding site (Fig. 7D), indicating that Blimp1 binds to the *Wnt5A* promoter. Expression of *Wnt5A* was significantly upregulated after *Blimp1* overexpression in DF1 cells (Fig. 7E), as was the level of H3K4me2 in the *Wnt5A* promoter (Fig. 7F). Interestingly, the level of LSD1 binding in the *Wnt5A* promoter was significantly downregulated (Fig. 7G). These results indicated that Blimp1 and LSD1 interact to regulate the expression of genes related to Wnt5A signaling.

Morphologic observation after interference with *Lsd1* and *Mll2* expression in the *in vitro* BMP4 induction model revealed that *Lsd1* interference via *Lsd1* shRNA (sh*Lsd1*) promoted PGC formation, whereas interference with *Mll2* expression inhibited PGC formation (Fig. S8A). Expression of genes that activate Wnt signaling – such as *Wnt5A*, β-catenin, *Fzd4* and *Tcf7l2* – increased significantly after interference using sh*Lsd1* (*P*<0.01), whereas expression of genes that suppress Wnt signaling, such as *Axin1* and *Apc*, decreased significantly (*P*<0.01) (Fig. S8B). Completely opposite results were obtained after interference with *Mll2* expression (Fig. S8B) and *in vivo* (Fig. S8C). Collectively, these data indicated that H3K4me2 regulates PGC formation by activating Wnt5A–β-catenin–TCF7L2 signaling.

### BMP4 initiates Wnt signaling to ensure normal PGC development

**Fig. 8.**
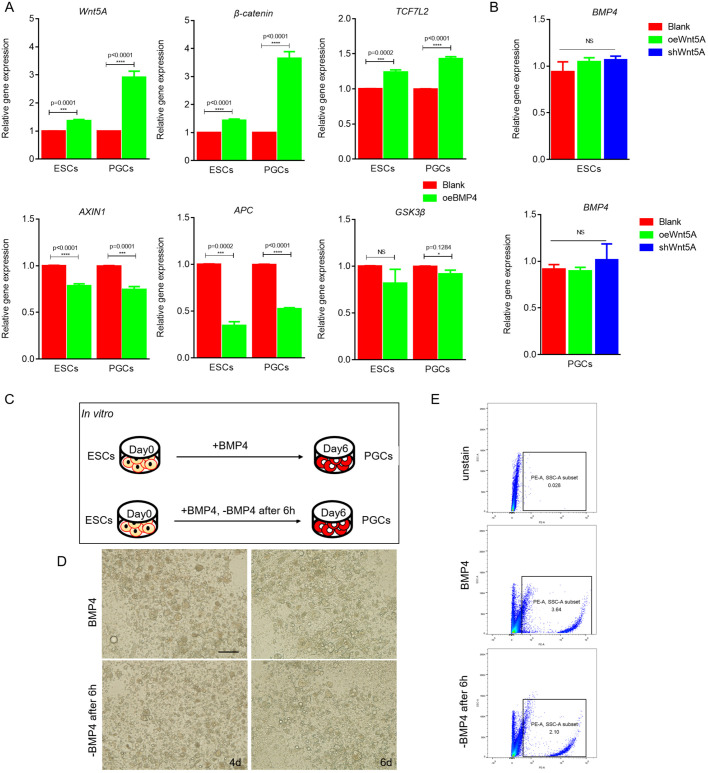
**BMP4 initiates Wnt signaling to ensure normal PGC development.** (A) qRT-PCR was used to detect the expression of Wnt signal-related molecules after BMP4 signal activation. (B) qRT-PCR was used to detect the expression of BMP4 signal-related molecules after Wnt signal activation in ESCs (top) and PGCs (bottom). (C,D) Cell morphology changes after BMP4 removal in the BMP4 model (C); images show morphological changes with BMP4 and BMP4 removal in the BMP4 model at 4 days (left column) and 6 days (right column) (D). Scale bar: 60 μm. (E) Flow cytometry analysis of PGC formation efficiency after BMP4 removal. The upper panel shows the negative control group, the middle panel shows the BMP4 induced group and the lower panel shows the BMP4 induced with BMP4 removal group. *P<0.05; **P<0.01; ***P<0.001; ****P<0.0001; NS, not significant (two-sample paired Student's *t*-tests).

As our collective results indicated that Wnt–*Lin28A*–*Blimp1*–Wnt functions as a positive-feedback loop during PGC formation, we sought to identify the factors that activate this feedback pathway. Previously, we confirmed that BMP4 plays an important role in PGC formation. We noticed that the addition of BMP4 protein for 6 h in ESC or PGC culture medium can significantly increase the expression of signal molecules such as *Wnt5A*, β-catenin and *Tcf7l2* (*P*<0.01) (Fig. 8A), and significantly downregulate the expression of genes such as *Axin1* and *Apc* (Fig. 8A), which preliminarily indicates that the BMP4 signal has an activating effect on the Wnt5A signal, and that BMP4/Smad proteins are upstream of the Wnt signal. After 6 h of *Wnt5A* overexpression and interference in ESCs and PGCs, the expression of *Bmp4* did not change significantly (Fig. 8B), indicating that the Wnt signal is downstream of the BMP4 signal. The function of both BMP4 and Wnt in PGC formation suggests that BMP4 activates downstream Wnt5A–β-catenin–TCF7L2 signaling to regulate PGC formation. Therefore, we preliminarily concluded that BMP4 signaling activates the Wnt–Lin28A–Blimp1–Wnt feedback system. To provide additional evidence, we changed the culture medium at 6 h (after Wnt signaling activation) during induction with BMP4 (Fig. 8C,D). Flow cytometry analysis revealed that the absence of BMP4 had no effect on formation of normal PGCs (Fig. 8E). Therefore, we concluded that BMP4 signaling mediates the normal development of PGCs by activating Wnt signaling.

## DISCUSSION

The present study established a new regulatory model for PGC formation. After activation of Wnt5A–β-catenin–TCF7L2 signaling, β-catenin competes with LSD1 to bind to TCF7L2 in the *Lin28A* promoter, leading to increased H3K4me2 levels and expression of *Lin28A*. Lin28A then activates the expression of *Blimp1* by inhibiting the maturation of *gga-let-7a-2-3p*, thus regulating PGC formation. Notably, *Blimp1* activates Wnt to initiate the Wnt–*Lin28A–Blimp1*–Wnt positive-feedback pathway (Fig. S8D).

Wnt signaling plays similar roles in the formation of mammalian and avian PGCs, but the regulatory mechanisms differ markedly (Cantú et al., 2016; Lee et al., 2016). The Wnt–β-catenin pathway promotes induction of the ectoderm response to BMP4 signaling and participates in determining PGC specialization in mammals (Aramaki et al., 2013; Yamaguchi et al., 1999), with Wnt directly regulating the expression of BLIMP1 and PRDM14 via mesoderm and notochord transcription factor T (Chassot et al., 2017; Ohinata et al., 2009; Saitou et al., 2005; Yamaguchi et al., 1999). This process differs significantly from that of chicken PGC development. Our research confirmed that Wnt signaling is involved in chicken PGC formation; the mechanism involves activation of *Lin28A* expression via Wnt signaling through TCF7L2 and indirect regulation of Blimp1 expression. The entire process is also regulated by H3K4me2, indicating that although Wnt signaling plays the same biological role in the formation of mammalian and chicken PGCs, its regulation differs by species.

Wnt signaling primarily affects target genes such as *c-myc* and *Jun* (He et al., 1998; Tetsu and McCormick, 1999). However, these genes play no obvious role in PGC formation. The present study confirmed that Wnt interacts with Lin28A, a key factor in PGC formation (Childs et al., 2012). Although many studies have examined the interaction between Wnt and Lin28A in mammals, primarily as it relates to cancer (McCarty, 2012; Oh et al., 2010; Pan et al., 2018), a role for this interaction in PGC formation has not been reported. Here, we confirm, for the first time, that *Lin28A* expression is directly regulated by Wnt signaling in chicken ESCs and PGCs, and our data provide new insights for studying the regulatory mechanism of mammalian PGC formation.

Wnt signaling activates downstream target genes through the β-catenin–TCF activation complex (Kelly et al., 2011; Schuijers et al., 2014; van Amerongen and Nusse, 2009) and regulates the chromatin status of target gene promoters by recruiting epigenetic factors that also regulate gene expression (Lee et al., 2006). In mammalian rectal cancer cells, activated Wnt signaling recruits histone methylation transferase via β-catenin, catalyzing the H3K4me3 modification in target gene promoters to regulate gene expression (Salz et al., 2014; Willert and Jones, 2006). In *Xenopus*, the recruitment of PRMT2 by β-catenin was used to establish the target gene promoter histone H3R8me2a modification that regulates the transcription of downstream genes during mesocotyl transition (Blythe et al., 2010). In addition to β-catenin, the TCF transcription factors also recruit histone methylation-modifying enzymes (Li et al., 2011). TCF4 regulates the transcription of classical Wnt signaling target genes by recruiting the histone-modification enzyme spindlin1 (Su et al., 2014). We found that Wnt–β-catenin–TCF signaling plays a unique regulatory role: activation of Wnt signaling leads to dissociation of the TCF7L2–LSD1 complex, thereby increasing H3K4me2 modification of the *Lin28A* promoter and activating *Lin28A* expression, thus promoting PGC formation.

Here, we confirmed that PGC formation is regulated by the Wnt–Lin28A–Blimp1–Wnt positive-feedback regulation system. BMP4 induces the production of Blimp1-positive cells in early blastocysts in both mammals and chickens (Saitou et al., 2005), but the underlying molecular mechanism has not been fully elucidated. We examined the mechanism of BMP4-induced Blimp1-positive cell formation. After activation of Wnt signaling by BMP4, *Lin28A* expression is activated by the transcription factor TCF7L2. Lin28A then activates Blimp1 expression by inhibiting the maturation of *gga-let-7a-2-3p*. Finally, activated Blimp1 feeds back the signal to further activate Wnt signaling. Thus, the entire process constitutes a positive-feedback regulatory system. Saitou et al. (2005) studied the process of mouse PGC formation and proposed that when BMP4 signaling is activated and Wnt signaling is suppressed, the expression of early PGCs marker genes such as *Blimp1* is reduced or completely suppressed. Activated Wnt signaling enhances the response of epiblasts to BMP4 signaling. The Wnt–Lin28A–Blimp1–Wnt positive-feedback regulation system proposed in this study reasonably explains this phenomenon.

## MATERIALS AND METHODS

### Reagents

Anti-H3K4me2 [ab32356; 10 μg for ChIP experiments, 1:2000 for western blotting (WB)], anti-histoneH3 (ab1791; 1:2000 for WB), goat anti-mouse IgG (ab6786), goat anti-rabbit IgG (ab6718), and rabbit anti-rat IgG (ab6730) were obtained from Abcam. Anti-Myc (14793; 1:50 for Co-IP, 1:1000 for WB) and anti-β-catenin (9587; 1:50 for Co-IP, 1:1000 for WB, 1:25 for ChIP) were obtained from Cell Signaling Technology. Anti-CVH [ab27591; 1:1000 for WB, immunohistochemistry (IHC) and fluorescence-activated cell sorting (FACS)] was obtained from Abcam.

### qRT-PCR

Total RNA was extracted from cells using Trizol reagent (Tiangen, Beijing, China) and reverse transcribed to synthesize cDNA using a Quantscript RT kit (Tiangen). Expression of Wnt-associated signaling molecules was assessed using β-actin as an internal control (Table S3). The qPCR reaction system (20 μl total volume) was as follows: 2 μl cDNA, 10 μl TB Green Premix Ex TaqII, 0.8 μl each of upstream and downstream primers (10 μM) and 6.4 μl ddH_2_O. PCR reaction procedures were carried out according to the instructions provided with the Takara TBGreen™ PremixExTaq™ II.

### Cell transfection

For the overexpression vector (carrier framework pCDNA3.0), 1.5×10^5^/well chick embryo fibroblasts (CEFs)/ESCs/PGCs were seeded into a 24-well plate. CEFs/ESCs/PGCs were seeded in 24-well plates (Corning) at 1×10^5^ cells/well. When cell density became 70%, cells were transfected with FuGENE^®^ HD (Promega) at a 3:1 ratio of reagent (volume, μl) to plasmid (mass, ng). It should be noted that in ESCs and PGCs, transfection should be conducted continuously three times (at 1 day intervals).

For interference vectors (lentiviral vector), 1×10^5^/well CEFs/ESCs/PGCs were seeded into a 24-well plate. When cell density became 70%, the lentiviral vector was used to infect CEFs/ESCs/PGCs under 10 multiplicity of infection and 5 ng/ml polybrene (Santa Cruz Biotechnology, sc-134220).

### Cell isolation and culture

#### ESCs

Chicken ESCs are derived from intracellular masses at the blastocyst stage. Fresh fertilized eggs were disinfected by 4% benzalkonium bromide and 75% alcohol. The blunt end of an egg was broken by tweezers and the egg white was removed, then blastoderm cells at stage X were collected by the spoon method in tissue culture dishes and rinsed in phosphate-buffered saline (PBS) to remove the yolks and vitelline membrane. After washing with PBS, ESCs were maintained in a 5% CO_2_ humidified atmosphere at 37.0°C with culture medium.

#### PGCs

Fertilized eggs at 4.5 days were sterilized with 4% benzalkonium bromide and 75% alcohol to obtain chicken embryos to separate the genital bridge. After the genital bridge was cut and digested by trypsin for 3 min, the cell suspension was obtained and filtered with 400 mesh filter cloth. The cell suspension was then centrifuged at 600 ***g*** for 6 min, the supernatant discarded and the cells collected. Cell precipitation was resuspended in PGC medium. KnockOut Dulbecco's modified Eagle medium (Gibco, A3181501) containing 10% fetal bovine serum, 2.5% chicken serum (Sigma-Aldrich, C5405), 100 U/ml penicillin, 100 U/ml streptolycin, 0.4μmol/l non-essential amino acid (Sigma-Aldrich, M7145), 2 mmol/l glutamine (Sigma-Aldrich, G7513), 0.1 mmol/l β-mercaptoethanol (Sigma-Aldrich, M6250), 10 ng/μl mouse leukemia inhibitory factor (Sigma-Aldrich, ESG1106), 10 ng/μl basic fibroblast growth factor (Sigma-Aldrich, GF446) and 5 ng/ml human stem cell factor (Sigma-Aldrich, GF021) was used to culture ESCs and PGCs.

### Vascular injection of chicken embryos and IHC

Embryos were collected from hatched eggs at 2.5 days (HH stage 13–17), into which a round hole with a diameter not exceeding 0.5 cm had been made using tweezers at the blunt end. The chicken embryo was exposed, and a micro-pipettor was used to inject the processed PGCs or the encased transfection vector into the embryonic blood vessel. The embryo was then cross-sealed with medical tape for further incubation. Embryos were collected at 4.5 days to prepare paraffin sections according to a previously reported procedure (Zhang et al., 2020). The paraffin sections were placed in pH 6.0, 0.01 M sodium citrate buffer solution, boiled in a microwave oven and allowed to cool to room temperature; this process was repeated four times. Then, the sections were incubated overnight at 4°C in anti-CVH primary antibody diluted in 5% bovine serum albumin, washed three times with PBS and incubated in secondary antibody for 1 h at room temperature, before further washing three times with PBS and application of 3,3′-diaminobenzidine (DAB)-H_2_O_2_ for 10 min. Mayer staining was then applied for 30* *s, before differentiation with hydrochloric acid and alcohol for 3* *s, immersion in running water for 15 min, and treatment with acetic acid for 2 min, ethanol for 2 min and xylene for 5 min. Sections were covered with neutral gum and observed under a microscope (Nikon).

All procedures involving the care and use of animals conformed to US National Institute of Health guidelines (NIH Pub. No. 85-23, revised 1996) and were approved by the Laboratory Animal Management and Experimental Animal Ethics Committee of Yangzhou University.

### Co-IP

DF1 cells (CEFs) and PGCs with good status were selected and divided into three groups for transfection. Cells were maintained in complete medium. One group was transfected with the overexpression (oe) β-catenin vector; one group was transfected with the oe*Tcf7l2*-*Myc* vector; and one group was transfected with the oeβ-catenin and oe*Tcf7l2-Myc* vectors. After culture at 37°C in 5% CO_2_ and saturation humidity for 48 h, the cells were collected for Co-IP experiments, as previously described (Zuo et al., 2018a).

### ChIP-qPCR

ESCs and PGCs with good status were selected and divided into three groups for transfection. Cells were transfected in factor medium. One group was transfected with sh*Lsd1*, one group was transfected with sh*Mll2*, and one group was left untreated. After incubation for 48 h at 37°C in 5% CO_2_ and saturation humidity, ChIP-qPCR was performed as follows: cell crosslinking and fragmentation, immunoprecipitation of crosslinked proteins/DNA, elution of protein/DNA complexes, and purification and recovery of DNA using centrifugal columns for ChIP-qPCR (Table S4).

### Wnt5A–β-catenin–TCF7L2 signaling target gene prediction

Binding target genes of the Wnt signaling transcription factor TCF7L2 in three different species (human, rat and mouse) were predicted using online software (http://gtrd.biouml.org/bioumlweb/#). GO functional annotation of the predicted target genes was carried out using DAVID (https://david.ncifcrf.gov/) and KOBAS (http://kobas.cbi.pku.edu.cn/kobas3/?t=1) to identify candidate genes related to germ cell development and stem cell differentiation.

### Analysis of *Lin28A* promoter activity

The 2000-bp genome sequence upstream and downstream of the coding sequence was identified based on the *Lin28A* sequence obtained from the National Center for Biotechnology Information (NCBI; https://www.ncbi.nlm.nih.gov/) and the University of California Santa Cruz (http://genome.ucsc.edu/). The promoter region and transcription start site were identified based on core promoter elements (TATA box, CAAT box and 5'-end of the coding region). Primers were designed with the transcription start site designated as +1 for amplification of fragments. The plasmid p*Lin28A*-EGFP was constructed and then used to transfect DF1 cells for 24–48 h until green fluorescence was observed under a fluorescence inverted microscope. The presence/absence of green fluorescence was used to confirm that the constructed promoter fragment exhibited promoter activity. The PGL3-basic vector of different deletion fragments of the *Lin28A* promoter was constructed for the dual luciferase reporter gene detection system, which was used to detect promoter activity of the *Lin28A* target gene. The protocol was as follows: recombinant plasmids encoding different promoter fragments were co-transfected into DF1 cells with pRL-SV40 at a mass ratio of 30:1. A negative control was simultaneously prepared (co-transfection of pGL3-basic and pRL-SV40 plasmids into DF1 cells at a mass ratio of 30:1). Detailed transfection methods are available from the FuGENE product manual. Three wells of cells were transfected for each group, and transfection was repeated three times. At 48 h after initial transfection, the cells were collected and 70 μl cell lysate was added to each tube and mixed gently. Next, 70 μl fluorescent solution was added to each well and gently mixed. Renilla fluorescence was measured using a fluorescent plate reader after addition of 70 μl STOP terminating reagent followed by gentle blowing, and mixing.

### Detection of *Lin28A* as a downstream target of Wnt5A/β-catenin

DF1 cells were transfected with the following vectors pcDNA3.1-β-catenin, pcDNA3.1-*Myc*-*Tcf7l2*, *Lin28A* promoter deletion, and the corresponding TCF7L2 binding-site mutation vector (pGL3.0-Basic+pcDNA3.1 served as the negative control). The change in promoter activity was assessed using the double luciferase reporter system, and the relative fluorescence activity is reported as the mean±s.e. of three experiments. ESCs and PGCs with good growth conditions were selected and divided into three groups each for transfection. Cells were transfected in factor medium. One group was transfected with shβ-catenin, one group was transfected with oeβ-catenin, and one group was left untreated. After incubation at 37°C and 5% CO_2_ for 48 h, ChIP-qPCR was performed.

### Role of *Lin28A* in PGC formation

ESCs were transfected with *Lin28A* overexpression and interference expression vectors and then either induced with BMP4 or injected into the blood vessels of chicken embryos. Cell samples were collected at 0, 2, 4 and 6 days after *in vitro* induction, and tissue samples were collected at 0 and 4.5 days during *in vivo* incubation. Total RNA was extracted using Trizol reagent, and cDNA was synthesized by reverse transcription. Expression of the PGC marker genes *Cvh*, *C-kit* and *Blimp1*, and the totipotent marker genes *Nanog* and *Oct4*, was analyzed using β-actin as an internal reference. qRT-PCR was conducted as previously described. The efficiency of PGC formation was assessed by flow cytometry and analysis of paraffin-embedded tissue sections.

### Assay of LSD1 binding to β-catenin and TCF7L2

DF-1 cells and PGCs with good status were selected, divided into six groups, and co-transfected with *Lsd1*, β-catenin or *Tcf7l2* vector at 37°C and 5% CO_2_ and saturation humidity for 48 h. Cells of each group were collected, and a lysate was prepared and subjected to immunoprecipitation (IP), after which the protein concentration was determined. Subsequent IP experiments were performed using an equal volume of lysate. Binding of LSD1 to β-catenin or TCF7L2 was confirmed by monitoring the expression of target protein (co-immunoprecipitated protein) by WB.

### Effect of LSD1 on β-catenin binding to TCF7L2

DF-1 cells and PGCs with good status were selected and divided into two groups on the basis of co-transfection with *Lsd1*-Flag and *Tcf7l2*-Myc. One group was co-transfected with β-catenin vector, and the other was co-transfected with pcDNA3.1 as a control. The cells were incubated at 37°C, 5% CO_2_ and saturation humidity for 48 h. Cells of each group were collected, and a lysate was prepared and subjected to IP, after which the protein concentration was determined. Subsequent IP experiments were carried out using an equal mass and volume of lysate, and expression of the co-precipitated target protein was evaluated by WB.

### *Lin28A*-targeted screening of *gga-let7* sequences

Online software (http://mirdb.org/cgi-bin/search.cgi) was used to predict chicken miRNA *gga-let7* sequences. DF1 cells, ESCs and PGCs with good status were selected and divided into three groups. One group was transfected with *Lin28A* siRNA (si*Lin28A*), one group was transfected with oe*Lin28A*, and one group was left untreated. DF1 cells were transfected in complete medium, and ESCs and PGCs were transfected in factor medium. After 48 h of incubation at 37°C and 5% CO_2_ and saturation humidity, total RNA was extracted using an miRNA extraction and isolation kit, and cDNA was synthesized by reverse transcription according to the reverse transcription kit procedure. U6 was used as an internal reference to detect changes in the relative expression of *let7* miRNAs. The *let7* miRNA quantitative primer sequences are shown in Table S5. qRT-PCR was carried out according to the instructions of the miRNA fluorescence quantitative detection kit. The reaction mixture was as follows: 50 ng cDNA, 10 μl 2× miRcute Plus miRNA PreMix, 0.4 μl forward primer, 0.4 μl reverse primer and ddH_2_O to a total volume of 20 μl. The PCR conditions were as follows: pre-denaturation at 95°C for 15 min, 94°C for 20* *s, 63°C for 30* *s, 72°C for 34* *s (five cycles); 94°C for 20* *s, 60°C for 34* *s annealing/extension (40 cycles); standard dissolution curve analysis.

### Screening and verification of *gga-let-7a-2-3p* target genes

Online software (http://mirdb.org/cgi-bin/search.cgi) was used to predict target genes related to reproductive differentiation. The targeting effect of *gga-let-7a-2-3p* on *Blimp1* was assessed using the dual luciferase reporter gene detection system. DF1 cells were co-transfected with *Blimp1*-3'UTR-WT or *Blimp1*-3'UTR-Mut and pRL-TK at a mass to volume ratio of 10:1. Based on this, *gga-let-7a-2-3p* mimics or inhibitors were added, and negative controls were set up at the same time. The protocol was as follows: 2×10^5^ DF-1 cells, PGCs and iPGCs/well were inoculated into a 24-well plate 1* *day in advance of the experiment and cultured without antibiotics. When cells reached 50–60% confluence, mimic (or inhibitor) was added and diluted with 50 μl Opti-MEM to a final concentration of 50 μM. *Blimp1*-3'UTR-WT (or the total mass of Blimp1-3'UTR-Mut cells was 1 μg) and pRL-TK were gently mixed as solution A, and 4 μl FuGENE HD was diluted with 50 μl Opti-MEM and gently mixed for 5 min at room temperature to serve as solution B. After mixing solutions A and B, the mixture was gently blown three to five times and left at room temperature for 20 min, before incubation at 37°C for 10–15 min. The mixture was then slowly added to the cell culture hole, mixed with 400 μl complete medium, and incubated at 37°C and 5% CO_2_. Three wells were transfected at a time, each transfection was repeated three times, and the cells were collected 48 h after transfection. Next, each tube was supplemented with 70 μl cell lysate and gently mixed, after which the same volume of fluorescent solution was added to each well. Renilla fluorescence was measured using a fluorescent plate reader after addition of 70 μl STOP terminating reagent followed by gentle blowing, and mixing. Values are reported as the mean±s.e. of three repeat tests. The assay was conducted according to the instruction manual of the dual luciferase reporter gene detection kit (Promega).

### Data analysis

Hierarchical clustering of differential gene expression (|log2| values) was performed using Heml. Relative gene expression was calculated from PCR data using the 2^−ΔΔCt^ method. The significance of between-group differences was assessed using two-sample Student's *t*-tests (paired) with SPSS software, version 18.0. Data are presented as the mean±s.d. unless otherwise indicated. Significance was set at *P*<0.05.

## Supplementary Material

Supplementary information

Reviewer comments
