# Supplementary material for: BMP4 activates the Wnt–Lin28A–Blimp1–Wnt pathway to promote primordial germ cell formation via altering H3K4me2
Source: J Cell Sci. 2021 Feb 1;134(3):jcs249375. doi: 10.1242/jcs.249375 (PMC7875490; doi:10.1242/jcs.249375)
Supplement: Supplementary information [file joces-134-249375-s1.pdf]

Supplementary Figures

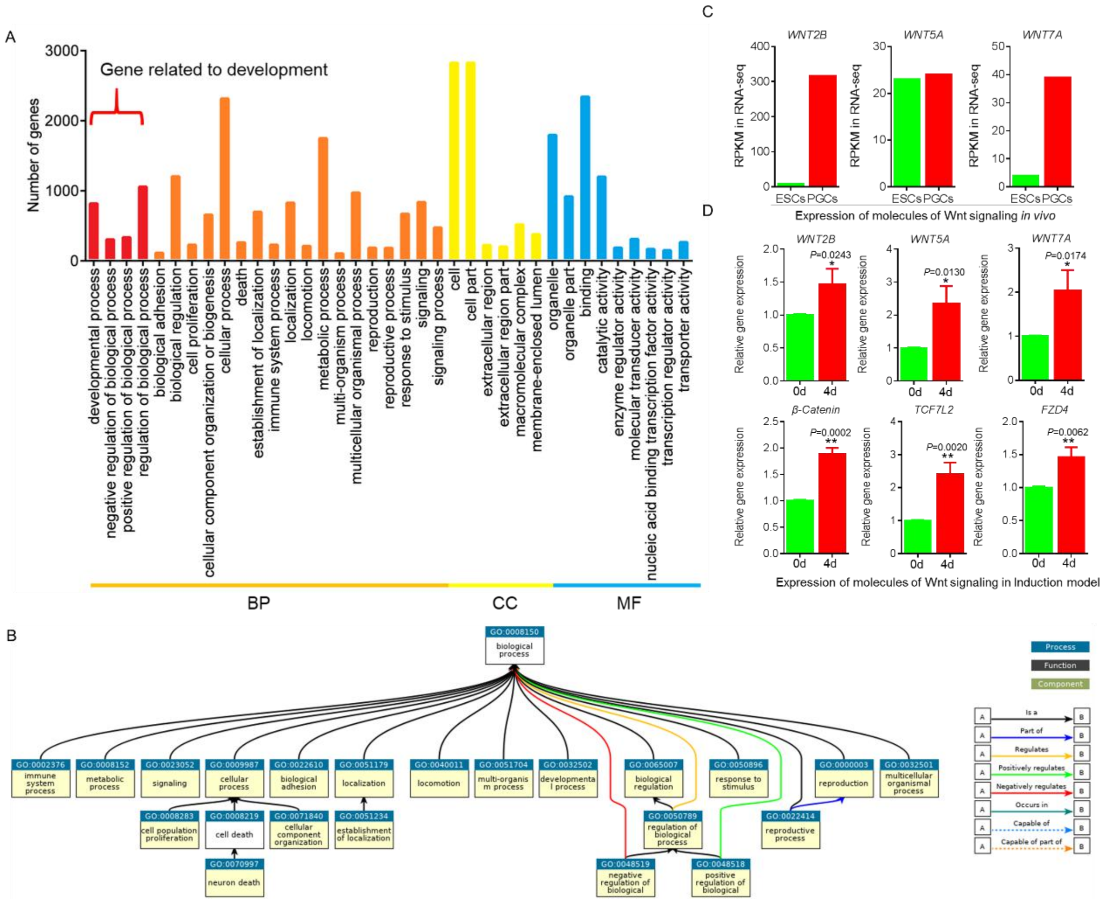

Figure S1. Screening of Wnt signaling pathway. A,B. GO analysis of DEGs (differentially expressed genes) in ESCs and PGCs and screened for development-related DEGs. C. RPKM of genes in Wnt signaling in ESCs and PGCs. D. Expression of genes of Wnt signaling in PGCs induction model *in vitro*.

Journal of Cell Science • Supplementary information

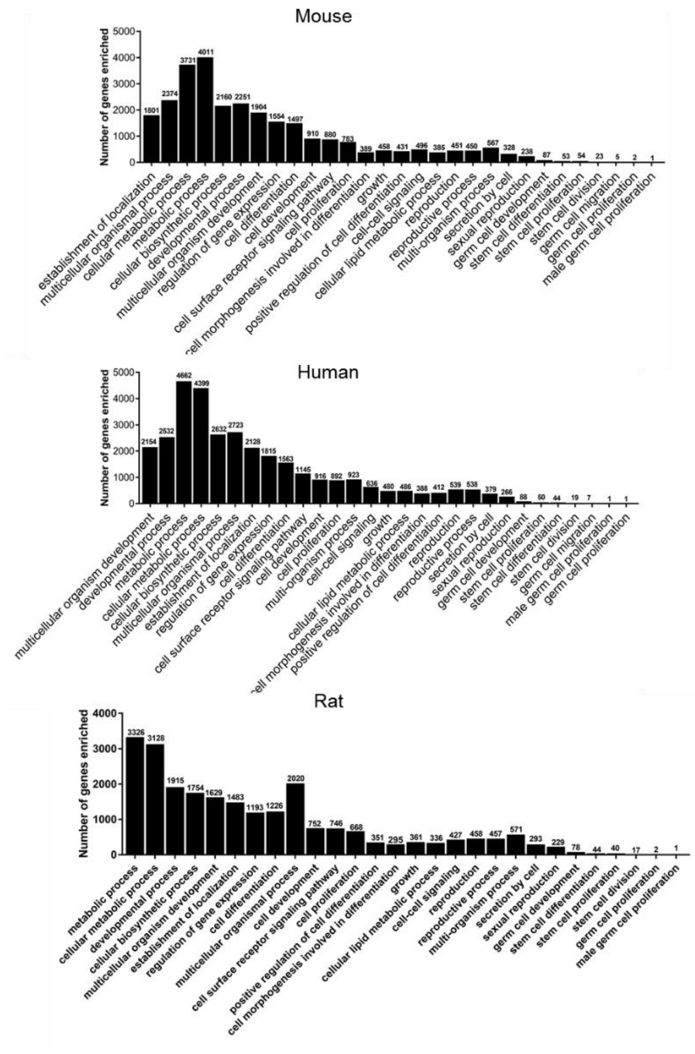

Figure S2. GO analysis of TCF7L2 target gene.

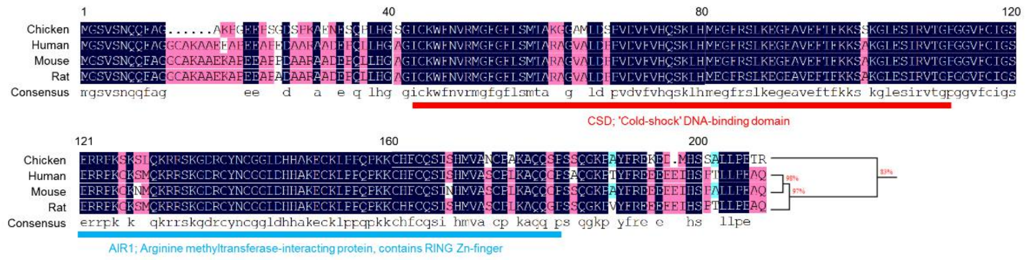

Figure S3. Conservation analysis of Lin28A protein in different species.

Journal of Cell Science • Supplementary information

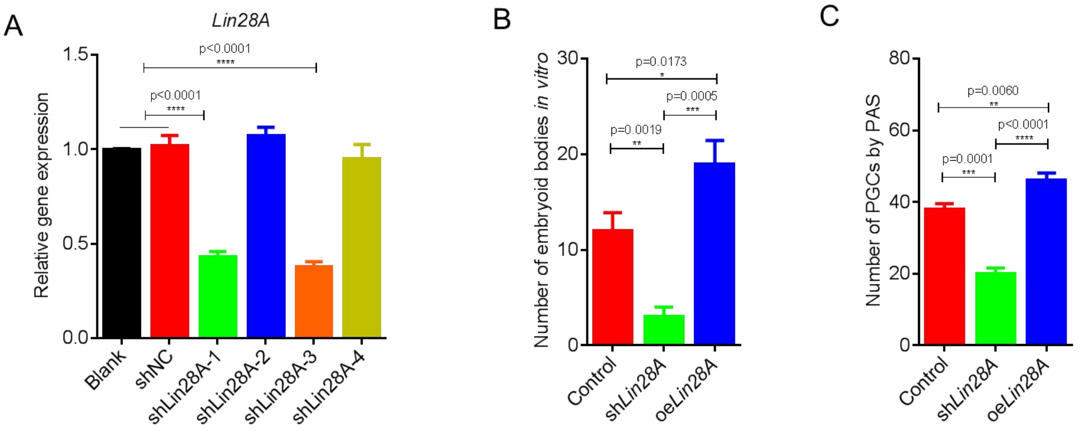

Figure S4. Lin28A regulates PGCs formation *in vitro* and *in vivo*. A. Activity detection of Lin28A interference expression vector. B. Changes in the number of embryoid bodies on D6 induced by different treatments. C, Number of PGCs in genital ridge was counted after Lin28A overexpression and interference *in vivo*.

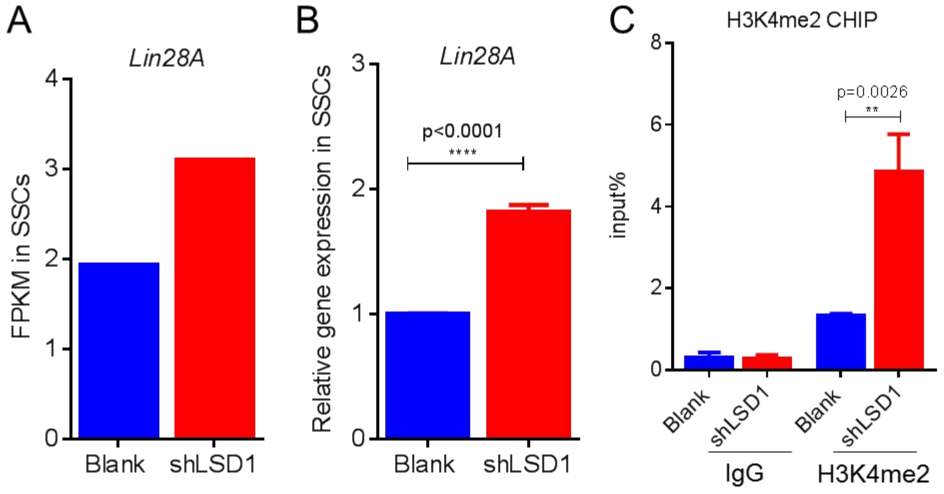

Figure S5. H3K4me2 regulates expression of Lin28A in SSCs marker genes. A. FPKM of Lin28A with different treatment in RNA-seq. B. Detection of Lin28A expression after interference with LSD1 in SSCs. C. Detection of H3K4me2 enrichment level in Lin28A promoter region after interfering with LSD1 in SSCs.

Journal of Cell Science • Supplementary information

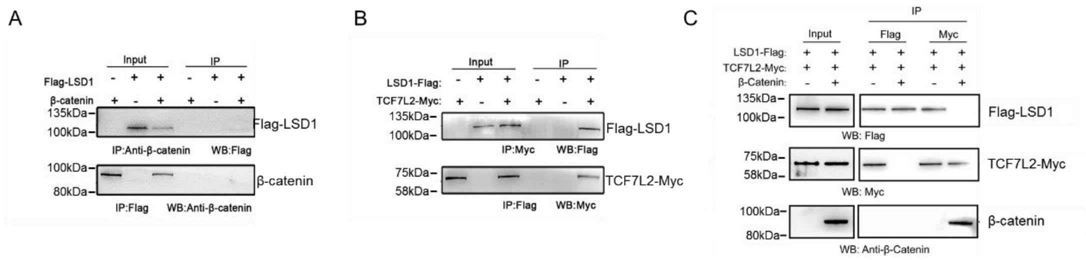

Figure S6. Results of Co-IP showed the interaction of LSD1, TCF7L2 and β-Catenin in DF-1.

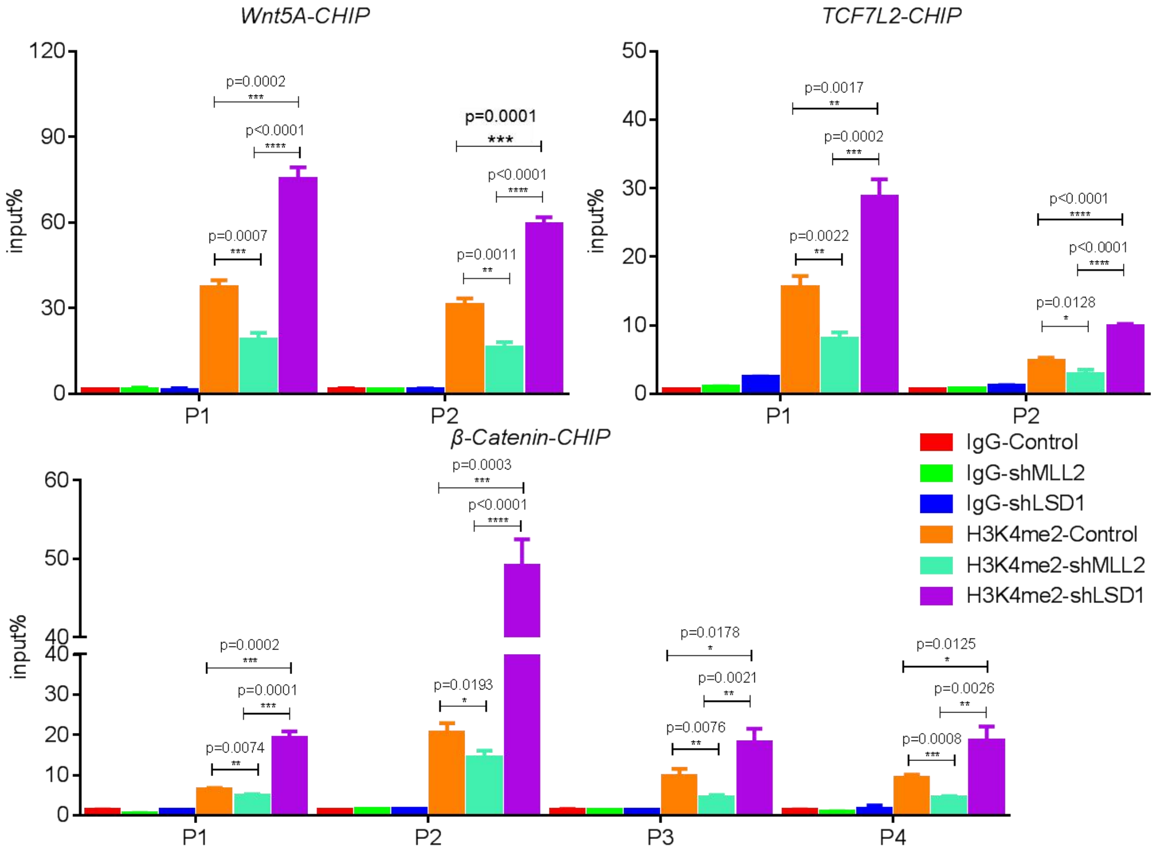

Figure S7. Detection of H3K4me2 enrichment in Wnt5A, β-Catenin, and TCF7L2 promoter regions after interference of LSD1 and MLL2 by CHIP-qPCR.

Journal of Cell Science • Supplementary information

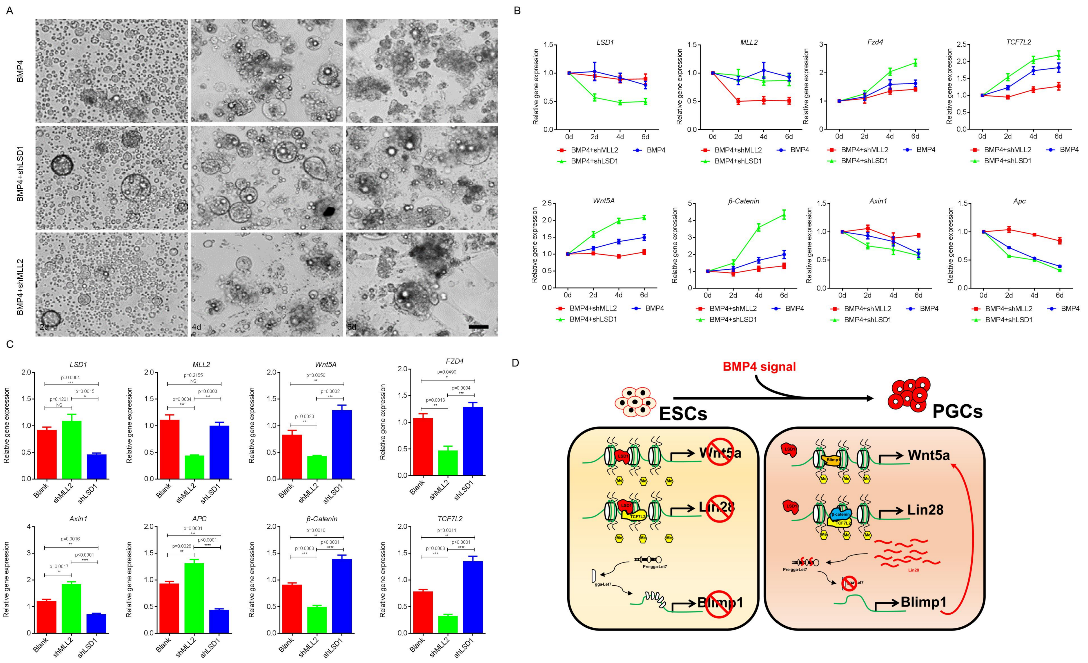

Figure S8. Detection of H3K4me2 enrichment in Wnt5A, β-Catenin, and TCF7L2promoter regions after interference of LSD1 and MLL2 by CHIP-qPCR. A. Changes in cell morphology after interference with LSD1 and MLL2 in the BMP4 induction model. Scale bar:100μm. B,C. After interfering with LSD1 and MLL2 *in vitro* and *in vivo*, the expression of Wnt signaling molecules was detected by qRT-PCR. D. A Wnt-Lin28-Blimp1-Wnt positive feedback regulation system was defined during the formation of PGCs, which was regulated by upstream BMP4 signals and H3K4me2.

Journal of Cell Science • Supplementary information

Table S1. 2516 DEGs in development-related GO terms

Click here to Download Table S1

Table S2. Expression of genes related to Wnt signaling pathway in RNA-seq

GeneID	Gene Name	ESCs - Expression	PGCs - Expression	Up-Down-Regulation (PGCs/ESCs)
XM_001233410	APC	1719	697	Down
NM_001012890	CTBP1	2622	5862	Up
NM_001039326	SOX17	2220	782	Down
NM_204295	CAMK2A	3	71	Up
XM_420494	DKK2	15	187	Up
NM_001030873	DVL1	427	1762	Up
NM_001030337	FZD1	1178	7182	Up
NM_204222	FZD2	758	6470	Up
NM_001031289	JUN	7073	62106	Up
XM_420551	MAPK10	2	56	Up
XM_417509	NFATC2	61	173	Up
XM_416036	PRICKLE1	2871	1241	Down
XM_422379	PRKACB	1255	3628	Up
XM_427339	SOST	1	17	Up
XM_421707	SFRP5	9	348	Up
NM_204783	WNT4	303	799	Up
NM_204336	WNT2B	9	316	Up
NM_204675	WNT3A	1062	0	Down
NM_204292	WNT7A	4	39	Up

Journal of Cell Science • Supplementary information

Table S3. qRT-PCR primers sequence of related genes

Gene		Primer Sequence（5'-3'）
Wnt5A	F:	AGGTGCTCTGGGGACACTT
	R:	TGGGGTTCATAGGGTTCATC
β-Catenin	F:	GGCAATCAAGAAAGTAAGC
	R:	AAGGTGGAGTCCTAAAGC
TCF7L2	F:	GCTGCGAAGAGGCAAGAT
	R:	CATCCTTGAGGGCTTGTCT
Fzd4	F:	ATTCCAATATGGCGGTGGAG
	R:	CGAGCACTTCTGCCATGTGT
LRP5	F:	AGGCCGAATTGCTCACCTAA
	R:	TGGCAATGCAGATGTGTGAG
APC	F:	GCAGCTCAGATTGCCAAGGT
	R:	TGGCTGCTCTCCTTGTGGTA
Axin1	F:	AGGACGAGGAGTGGAAAT
	R:	TCCCGTGTTGACATAGTAAGG
LSD1	F:	GCGATGGTGGTAACAGGACT
	R:	AACAGCTTGTCCGTTTGCTT
MLL2	F:	TCCCCGAGGGGATGACAG
	R:	TGCACGTCTTTGCTCTCCAT
Lin28A	F:	GGCGTCTTCTGCATTGGC
	R:	TGGCGACCATGTGGCTGA
Lin28B	F:	CGGTTGATGTCTTTGTGC
	R:	CCTTTGGGTCGTCTTTCA
Nanog	F:	CGTCCTACGGCTCTGTTA
	R:	CCTTCCTTGTCCCACTCT
Cvh	F:	AGGAGGACTGGGACACG
	R:	GCCTCTTGATGCTACCG
C-kit	F:	GCGAACTTCACCTTACCCGATTA
	R:	TGTCATTGCCGAGCATATCCA
Blimp1	F:	ATGAAGGCTGCTACACGG
	R:	GCAGTTTGATGCGTATTTG
β-actin	F:	CAGCCATCTTTCTTGGGTAT
	R:	CTGTGATCTCCTTCTGCATCC

Journal of Cell Science • Supplementary information

Table S4. ChIP-qPCR related primers

ChIP-qPCR		Primer（5’ -3'）
Lin28A-P1	F:	ATCCCGCGTGCCGCAATT
	R:	TTAGGGGCGAGGCCACCT
Lin28A-P2	F:	TTGACAGGTGGCCTCG
	R:	GACTCCATCTTCACTCCAACAA
Lin28B-P1	F:	GAGCCGATAACATCTGACA
	R:	TGCTCAAGCCCATCAAAT
Lin28B-P2	F:	ACTTCCAGCCAGTGTTGTG
	R:	TACTTCGGCGAGCGGCTTCCTG
Lin28B-P3	F:	CGGAGAAACCTGTGAAGC
	R:	TTTCGGCAGCTCACCCAC
β-Catenin-P1	F:	TCTCCCTACTTTGTTGTC
	R:	CAGCCAGAAGGTGGATGA
β-Catenin-P2	F:	GCTGTTTGTTACCACCCG
	R:	TGTTCATTTGCCTCCTAC
β-Catenin-P3	F:	TCACCACTGCCTTGAAAC
	R:	GAGATGGGAGGTACTCAA
β-Catenin-P4	F:	TTCTGAGGGCATCCATAC
	R:	TGCCCAAATGTTCTTCTC
Wnt5A-P1	F:	GGGGATAATTGAAATGCCACA
	R:	GGTTCGCTCCTCGGGTAA
Wnt5A-P2	F:	CGCTGCTCTGATAATGTCCC
	R:	CCTCCTTCTCCAGCGTCTTT
Tcf7l2-P1	F:	GCGGCACAGGCTCCCAATA
	R:	CCAGTGACTTTGGGCTTC
Tcf7l2-P2	F:	ATACGGCTGTTTGAGTTT
	R:	AACGCCTGCCTGCTGCTAC

Journal of Cell Science • Supplementary information

Table S5. qRT-PCR primers sequence of let7s

Name	Primer（5’ -3'）
gga-let-7a-2-3p	GCCGAGCTGTACAACCTCCTAGCTTTCC
gga-let-7a-3p	GCCGAGCTATACAATCTACTGTCTTTCC
gga-let-7b	GCCGAGTGAGGTAGTAGGTTGTGTGGTT
gga-let-7c-3p	GCCGAGCTGTACAACCTTCTAGCTTTCC
gga-let-7c-5p	GCCGAGTGAGGTAGTAGGTTGTATGGTT
gga-let-7d	GCCGAGAGAGGTAGTGGGTTGCATAGT
gga-let-7f-3p	GCCGAGCTATACAATCTATTGCCTTCCC
gga-let-7f-5p	GCCGAGTGAGGTAGTAGATTGTATAGTT
gga-let-7g-3p	CTGTACAGGCCACTGCCTTGCC
gga-let-7g-5p	GCCGAGTGAGGTAGTAGTTTGTACAGT
gga-let-7i	GCCGAGTGAGGTAGTAGTTTGTGCTGT
gga-let-7j-3p	GCCGAGCTATACAGTCTATTGCCTTCCT
gga-let-7j-5p	GCCGAGTGAGGTAGTAGGTTGTATAGTT
gga-let-7k-3p	GCCGAGCTATACAATCTACTGTCTTTCC
gga-let-7k-5p	GCCGAGTGAGGTAGTAGATTGAATAGTT
gga-let-7l-3p	CGATGCAGCCGACTACTTTCC
gga-let-7l-5p	GCCGAGTGAGGTAGTCGGTTGTATTGTT
U6	F:TAAGCCTGGACTGAGTAAGAGCG<br>R:CCATATTAGAAGCCCCTTTTTGT

Journal of Cell Science • Supplementary information
