# Supplementary material for: BMP4 activates the Wnt–Lin28A–Blimp1–Wnt pathway to promote primordial germ cell formation via altering H3K4me2
Source: J Cell Sci. 2021 Feb 1;134(3):jcs249375. doi: 10.1242/jcs.249375 (PMC7875490; doi:10.1242/jcs.249375)
Supplement: Reviewer comments [file joces-134-249375_review_history.pdf]

BMP4 activates the Wnt-*Lin28A-Blimp1*-Wnt pathway to promote primordial germ cell formation via altering H3K4me2

Qisheng Zuo, Kai Jin, Man Wang, Yani Zhang, Guohong Chen and Bichun Li
DOI: 10.1242/jcs.249375

Editor: John Heath

Review timeline

Original submission:	26 May 2020
Editorial decision:	28 July 2020
First revision received:	1 October 2020
Editorial decision:	27 October 2020
Second revision received:	10 December 2020
Accepted:	11 December 2020

Original submission

First decision letter

MS ID#: JOCES/2020/249375

MS TITLE: BMP4 signaling activates the Wnt-Lin28A-Blimp1-Wnt positive feedback pathway to promote primordial germ cell formation via altering H3K4me2

AUTHORS: Qisheng Zuo, Kai Jin, Yani Zhang, Guohong Chen, and Bichun Li
ARTICLE TYPE: Research Article

We have now reached a decision on the above manuscript.

To see the reviewers' reports and a copy of this decision letter, please go to: https://submit-jcs.biologists.org and click on the 'Manuscripts with Decisions' queue in the Author Area. (Corresponding author only has access to reviews.)

As you will see, the reviewers raise a number of substantial criticisms that prevent me from accepting the paper at this stage. They suggest, however, that a revised version might prove acceptable, if you can address their concerns. If you think that you can deal satisfactorily with the criticisms on revision, I would be pleased to see a revised manuscript. We would then return it to the reviewers.

We are aware that you may currently be unable to access the lab to undertake experimental revisions. If it would be helpful, we encourage you to contact us to discuss your revision in greater detail. Please send us a point-by-point response indicating where you are able to address concerns raised (either experimentally or by changes to the text) and where you will not be able to do so within the normal timeframe of a revision. We will then provide further guidance. Please also note that we are happy to extend revision timeframes as necessary.

Please ensure that you clearly highlight all changes made in the revised manuscript. Please avoid using 'Tracked changes' in Word files as these are lost in PDF conversion.

I should be grateful if you would also provide a point-by-point response detailing how you have dealt with the points raised by the reviewers in the 'Response to Reviewers' box. Please attend to

© 2020. Published by The Company of Biologists under the terms of the Creative Commons Attribution License (https://creativecommons.org/licenses/by/4.0/).

all of the reviewers' comments. If you do not agree with any of their criticisms or suggestions please explain clearly why this is so.

Reviewer 1

Advance summary and potential significance to field

In the manuscript entitled “BMP4 signaling activates the Wnt-Lin28A-Blimp1-1 Wnt positive feedback pathway to promote primordial germ cell formation via altering H3K4me2”, Zuo et al. continued the long term effort in charactering chicken germ cell formation in Li lab, and discovered a scientifically significant and potentially applicable pathway that determines avian primordial germ cell formation and may be useful for endanger avian species conservation.

Comments for the author

I suggest that the authors may consider down play the application part in the introduction and bring some of the cross-species comparison listed . One very powerful feature of avian PGCs is that they can be cultured long term in vitro especially for chicken PGCs. It is therefore awkward to suggest in vitro reprogramming or transdifferentiation of germ cells from other cell lineages without even discuss and compare to PGC culture. Also, introducing PGCs from ESCs from endangered species would be particularly impractical (the work from chicken fibroblasts, DF1, may be more relevant. But still, the cross-species differences may be too big). I think this story has enough scientific merit by introducing the molecular interaction and mechanism for PGC modulation, there is no need to push too much for application.

The authors tackled the rather complicated relationship among various known and new players of germ line specification and modulation in chicken and beyond including but not limited to BMP4, Wnt5a, beta-catenin, TCH7L2, miR-let7s, Lin 28A, Blimp1, CVH (VASA) as well as epigenetic modulators LSD1 and MLL2. While most of the factors are familiar to germ cell biologists, I do not think this would apply to the general reader of JoCeS. I think this manuscript seriously needs either a graphic summary or working model figure, to orient the readers of the rather complex relationship for the binding and regulating actives among these important factors over epigenetic modulation and beyond.

Also, in the abstract, I think the authors need to provide a little bit more concise background introduction to help the readers follow their story line.

Figure 1 and the associated opening text in the result session made the entry of Wnt and associated pathway that the authors focused on in this manuscript, by describing the features of differentially expressed genes (DEGs) between chicken embryonic stem cells and PGCs. However, I could not easily find where they got the list of DEGs. Was the from their previous studies or from public domain database?
Either way is fine but please cite the reference or upload their own datasets to GEO database and get a GSE number. Similarly, please kindly check whether all datasets mentions din the manuscript (with various knockout sets as well as over expressions) have been properly cited, in order to maximize the impact of the current study in case the readers would like to perform their own analysis based on theses resources.

I am not sure about the policy of JoCeS but it seems that this article needs an accompanying abbreviation list to facilitate readability.

Line 212 Not very clear, please consider rephrasing. Do you mean “which micRNA-let7” interacts with Lin28A?

-Also, since they are observing the effect of over expression/ knock down specific gene during the bmp4 induction in forming PGC from esc, I think they should show some data to support the success of the induction, maybe the similar expression profile of their successful induced PGC in this paper to their previous paper's FACS sorted PGC’s RNAseq data.

© 2020. Published by The Company of Biologists under the terms of the Creative Commons Attribution License (https://creativecommons.org/licenses/by/4.0/).

About the co-IP experiments illustrated in Fig. 1D and 1E, no methods was mentioned of how they transfected the cells: df1, esc, or induced pgc (since we think the transfection efficiency is really low in cPGC in our hands, I am interested about how they transfect the cells, and how was the transfection efficiency).

It seems that the co-IP experiment should be done in the induced PGC instead of df1(fibroblast) to directly support TCF7L2 and βcatenin's interaction during the differentiation of ESCs into PGC.

I am interested in which CVH(vasa) antibody the authors used. It might be the same as their previous paper https://onlinelibrary.wiley.com/doi/pdf/10.1002/jcb.26329
? No information was mentioned in the materials and methods. Also I can't find their antibody by their materials in previous paper.

-the previous paper they published and cited: 25 in (Fig. 1E)
https://onlinelibrary.wiley.com/doi/pdf/10.1002/jcb.26329 has nothing to deal with TCF7L2 , and merely by co-IP of β-catenin/TCF7L2 in chicken fibroblast(df1) may not be enough to conclude "Wnt5a-β-catenin-TCF7L2 positively regulates PGC formation" . More direct evidence may be needed to support.

Most of the experiments using overexression/ knockdown of specific gene in analyzing its effect on inducing PGCs the gain/loss of function assays are logically sound, but the co-ip experiment are conducted and mirn agga-let-7a-2-3p (mimic and inhibitor with Blimp1-3'UTR-WT and Blimp1-3'UTR-Mut.) are conduct in chicken fibroblast(df-1), I think they should be done in induced PGC or at least change their wording .

Reviewer 2

Advance summary and potential significance to field

The authors have elucidated several steps of the wnt pathway involved in PGC differentiation in the chicken. These data would be helpful for future studies of PGC derivation from ESC.

Comments for the author

The present paper investigates the molecular mechanisms by which BMP4 and Wnt regulate PGC differentiation in the chicken.
Using both in vitro and in vivo approaches authors present a large number of results and the overall conclusions seem appropriate although the wealth of data does not always facilitate the deduction of a clear result and how it has been obtained.
8 figures and 8 supplementary figures, each containing at least 5 panels cause confusion that should be cleared up. Some data have been previously published by the same authors or others. The new data should be presented more concisely. In other words results should be shortened and their interpretation should be moved to discussion section.

It is unclear how PGC were isolated.

It is unclear the source of ESC.

Provide more details about how the in vivo experiments were performed.

First revision

Author response to reviewers' comments

Dear Editor and Reviewers:

© 2020. Published by The Company of Biologists under the terms of the Creative Commons Attribution License (https://creativecommons.org/licenses/by/4.0/).

Thank you for your time and effort in dealing with our manuscript “BMP4 signaling activates the Wnt-Lin28A-Blimp1-Wnt positive feedback pathway to promote primordial germ cell formation via altering H3K4me2” (*JOCES/2020/249375*) in *Journal of Cell Sicence*. We are very pleased to see these comments, which are constructive and helpful to improve the quality of our study. Based on this, we carefully revised the manuscript and addressed all the comments raised by the reviewers point by point. We hope that this vision of our manuscript will meet the requirements for *Journal of Cell Sicence* publications.

We have addressed all the questions. The response was highlighted in red following each comment.

Reviewer 1 Comments for the Author:

Reviewer 1-1 I suggest that the authors may consider down play the application part in the introduction and bring some of the cross-species comparison listed . One very powerful feature of avian PGCs is that they can be cultured long term in vitro, especially for chicken PGCs. It is therefore awkward to suggest in vitro reprogramming or transdifferentiation of germ cells from other cell lineages, without even discuss and compare to PGC culture. Also, introducing PGCs from ESCs from endangered species would be particularly impractical (the work from chicken fibroblasts, DF1, may be more relevant. But still, the cross-species differences may be too big). I think this story has enough scientific merit by introducing the molecular interaction and mechanism for PGC modulation, there is no need to push too much for application.

Thank you for your suggestion. We have made a lot of modifications in the ‘Introduction’ part. In the new ‘Introduction’, we mainly compared the differences of PGCs formation mode and regulation mechanism among different species, and reduce the description of PGCs application. details are as follows**(Line46-68)**: primordial germ cells (PGCs) are the progenitor of sperm and ovum, which are responsible for the transmission of genetic information between germ lines.(Liu et al., 2018; Nakamura, 2017). Chicken PGC with characteristic of migrating to the genital ridge via the blood ensures its wide application in recovery of endangered bird species, gene editing and the generation of transgenic birds (Lu et al., 2014; Ono and Machida, 1999; Zhao and Kuwana, 2003). However, the limited number of PGCs greatly limits its application (Chen et al., 2020; Tajima et al., 1998; Yasuda et al., 1992). The lack of understanding of the molecular mechanisms of PGCs formation in chickens makes it difficult to obtain a large number of available PGCs in *vitro* via culture and induction, although the molecular mechanisms of PGCs formation in mammals are well studied.

Presently, the origin of PGC can be divided into two theories: Epigenesis and Preformation (Lasko and Ashburner, 1988; Wentworth et al., 1989). The formation of PGC in mammals belongs to Epigenesis. During the E5.5-E6.25 stage of mouse embryo development process, Part of the mesoderm cells induced by the BMP4 signal secreted by the ectoderm, began to express *Blimp1, Prdm1, Tcfap2c* et al, and finally differentiated into PGC, then migrated from the bottom of the allantoic sac to the endoderm and finally settled in the genital ridge(Saitou and Yamaji, 2012; Yamaguchi et al., 2000). The formation of PGC in invertebrates such as drosophila belongs to preformation. The origin of PGC in drosophila is influenced by germplasm RNA (maternal genetic factor). Within 1.5 to 3h of laying eggs, About 10 PGC precursors already exist in the embryo backend of drosophila, which is completely different from that of mammals(Spudich et al., 1998). Chicken PGC originates from the ectoderm, then gradually transfers to the endoderm. Finally Chicken PGC migrated to the genital ridge (HH10-12 Stage) through blood(Aige-Gil and Simkiss, 1991; Vick et al., 1993). The formation of PGCs in chickens is different from that in drosophila and mammals, and which theory it belongs to needs further exploration.

Reviewer 1-2 The authors tackled the rather complicated relationship among various known and new players of germ line specification and modulation in chicken and beyond, including but not limited to BMP4, Wnt5a, beta-catenin, TCH7L2, miR-let7s, Lin 28A, Blimp1, CVH (VASA) as well as epigenetic modulators LSD1 and MLL2. While most of the factors are familiar to germ cell biologists, I do not think this would apply to the general reader of JoCeS. I think this manuscript seriously needs either a graphic summary or working model figure, to orient the readers of the rather complex relationship for the binding and regulating actives among these important factors over epigenetic modulation and beyond.

Thank you for your suggestion. In this revision, we have provided a clear graphical abstract as below(Journal of Cell Science does not publish graphical abstracts, therefore, it was supplied in this

© 2020. Published by The Company of Biologists under the terms of the Creative Commons Attribution License (https://creativecommons.org/licenses/by/4.0/).

file):

Figure 9 [We have removed a figure which was provided to the reviewers in confidence]

Reviewer 1-3 Also, in the abstract, I think the authors need to provide a little bit more concise background introduction to help the readers follow their story line.

Thank you for your suggestion. We redescribe the background in the abstract, which makes it easier for the reader to understand the content of the manuscript. Details are as follows(**Line24-30**):

The unique developmental characteristics of Chicken primordial germ cells (PGCs) enable it to be used in recovery of endangered bird species, gene editing and the generation of transgenic birds, but the limited number of PGCs greatly limits its application. Studies have shown that the formation of mammalian PGCs is induced by BMP4 signal, but the formation mechanism of chicken PGCs has not been determined.

Reviewer 1-4 Figure 1 and the associated opening text in the result session made the entry of Wnt and associated pathway that the authors focused on in this manuscript, by describing the features of differentially expressed genes (DEGs) between chicken embryonic stem cells and PGCs. However, I could not easily find where they got the list of DEGs. Was the from their previous studies or from public domain database? Either way is fine but please cite the reference or upload their own datasets to GEO database and get a GSE number. Similarly, please kindly check whether all datasets mentions din the manuscript (with various knockout sets as well as over expressions) have been properly cited, in order to maximize the impact of the current study in case the readers would like to perform their own analysis based on theses resources.

Thank you for your suggestion. In order to describe the process of obtaining the DEGs(ESCs and PGCs) and the screening process of Wnt signaling pathway, we reintegrated the results section and provided specific DEGs data, and we supplied the SRR number(**SRR3720923 and SRR3720924, SRR database just as GEO database for RNA-seq in NCBI**). Details are as follows(**Line98-104**):

In order to analyze the molecular mechanism regulating chicken PGCs formation, DEGs (Differentially expressed genes) of ESCs and PGCs was obtained by RNA-seq (Fig. 1A~C, Supplementary Fig.1A,B), and annotated the function by GO (Gene Ontology). The results showed that 2516 DEGs were enriched in development-related GO terms(Supplementary Fig. 1A,B, Supplementary Table 1), and the KEGG pathway enrichment of which revealed that 20 genes were significantly enriched in the Wnt signaling pathway (P=0.0023)(Fig. 1C, Supplementary Table2 and Supplementary Fig. 1C).

In addition, we carefully examined the relevant data in the manuscript, the data related to the overexpression and interference of Wnt5a, and the data related to the interference of LSD1 and MLL2, have been published in our previous results, and we have supplied the corresponding references in manuscript.

We have submitted the RNA-seq data of SSCs interference with LSD1 to the public database, and we have provided the GSE number in the manuscript(GSM4771929 and GSM4771930)(**Line555- 557**).

Reviewer 1-5 I am not sure about the policy of JoCeS but it seems that this article needs an accompanying abbreviation list to facilitate readability.

Thank you for your suggestion. We provide the abbreviations as follows(**Line559-560**): primordial germ cells :PGCs; Embryonic stem cells: ESCs;Gene Ontology:GO; Co immunoprecipitation:Co-IP; embryoid bodies: Ebs; Periodic acid-Schiff staining: PAS.

Reviewer 1-6 Line 212 Not very clear, please consider rephrasing. Do you mean “which micRNA- let7” interacts with Lin28A?

Thank you for your suggestion. We redescribed the original sentence to make the meaning clearer. Details are as follows(**Line225-232**):

To identify specific *micRNA-let7* interacting with *Lin28A*, The expression of these mature *micRNA- let7s*

© 2020. Published by The Company of Biologists under the terms of the Creative Commons Attribution License (https://creativecommons.org/licenses/by/4.0/).

in chicken ESCs and PGCs was evaluated by qRT-PCR after *Lin28A* overexpression/interference, the results indicated that *gga-let-7a-2-3p* was significantly regulated by *Lin28A* in ESCs and PGCs. *gga-let-7a-2-3p* was significantly up-regulated following *Lin28A* overexpression and significantly down-regulated following *Lin28A* interference. Combined with previous studies, we can draw a conclusion that *gga-let-7a-2-3p* of *micRNAlet7s* can interact with *Lin28A* during the formation of PGCs in chickens.

Reviewer 1-7 Also, since they are observing the effect of over expression/ knock down specific gene during the bmp4 induction in forming PGCs from ESCs, I think they should show some data to support the success of the induction, maybe the similar expression profile of their successful induced PGCs in this paper to their previous paper's FACS sorted PGCs's RNAseq data.

Thank you for your suggestion. Our team successfully established the model of chicken ESCs differentiation into PGCs induced by BMP4 *in vitro*. In previous publications, we have demonstrated the feasibility of the induction model by flow cytometry（Zuo et al., Food and function, 2018 ）. In our another manuscript (*Nature Communicaton*, revision), which also involve the induction model. In the manuscript, we systematically prove that BMP4 can induce ESCs into PGCs *in vitro* by RNA-seq, The analysis results showed that the induced iPGCs and the primary PGCs had similar expression profiles. **[We have removed a figure which was provided to the reviewers in confidence]**

Figure RNA-seq analysis of ESCs, PGCs and iPGCs. A. PCA analysis of ESCs, PGCs and iPGCs. B. heatmape of ESCs, PGCs and iPGCs. C. Expression of PGCs related genes in three types of cells, which were highly expressed in PGCs and iPGCs, but low in ESCs.

Reviewer 1-8 About the co-IP experiments illustrated in Fig. 1D and 1E, no methods was mentioned of how they transfected the cells: df1, esc, or induced pgc (since we think the transfection efficiency is really low in cPGCs in our hands, I am interested about how they transfect the cells, and how was the transfection efficiency).

Thank you for your suggestion. As you said, as stem cells, ESCs and PGCs do have low transfection efficiency. In our study, Fugene was selected as the transfection reagent for the transfection of stem cells (Fugene can ensure the high transfection efficiency of the exogenous vector in stem cells). In order to ensure the normal operation of the experiment, we designed different transfection strategies for different cells. Details are as follows(**374-384**): Cell transfection

For Overexpression vector (The carrier framework is pCDNA3.0): 1.5×10^5/well CEF/ESCs/PGCs were seeded into a 24-well plate. CEF/ESCs/PGCs were seeded in 24-well plates (Corning, NY, USA) at 1 × 10^5 cells/well. When cell density became 70%, cells were transfected with FuGENE® HD (Promega, USA) at a 3:1 ratio of reagent (volume:ul) to plasmid(mass:ng). It should be noted that in ESCs and PGCs, transfection should be conducted continuously for 3 times(each interval is 1 day).

For interference vectors (lentiviral vector): 1×10^5/well CEF/ESCs/PGCs were seeded into a 24-well plate. When cell density became 70%, the lentiviral vector was used to infect CEF/ESCs/PGCs under 10 multiplicity of infection and 5ng/ml polybrene (Santa Cruz, California, USA, SC-134220).

Reviewer 1-9 It seems that the co-IP experiment should be done in the induced PGCs instead of df1(fibroblast) to directly support TCF7L2 and ßcatenin's interaction during the differentiation of ESCs into PGCs.

Thank you for your suggestion. We carried out the co-IP experiment in PGCs(Fig.1E), which made the results more convincing. Meanwhile, we explained the reasons for the experiment in DF-1: Firstly, we have confirmed that TCF7L2 and β-catenin are highly expressed in PGCs through RNA- seq data and qRT-PCR. Secondly, due to the lack of antibody of TCF7L2, β-catenin for chicken in the current market, it is difficult to carry out co-IP experiment using antibodies directly. In addition, the label fusion expression vector of TCF7L2 and β-catenin overexpressed in DF-1 can not only simulate the environment in PGCs, but also complete the relevant IP experiment through label antibody.

In order to ensure the reliability of the experimental results, we used the IP results in PGCs to replace the original experimental results in Fig.1E.

© 2020. Published by The Company of Biologists under the terms of the Creative Commons Attribution License (https://creativecommons.org/licenses/by/4.0/).

Reviewer 1-10 I am interested in which CVH(vasa) antibody the authors used. It might be the same as their previous paper https://onlinelibrary.wiley.com/doi/pdf/10.1002/jcb.26329 ? No information was mentioned in the materials and methods. Also I can't find their antibody by their materials in previous paper.

Thank you for your suggestion. The CVH(Vasa, DDX4) Antibody from abcam (Ab27591). The antibody is a mouse antibody, which has been verified by in our previous study to be used to label CVH protein in chicken PGCs(**Line364**).

Reviewer 1-11 the previous paper they published and cited: 25 in (Fig. 1E) https://onlinelibrary.wiley.com/doi/pdf/10.1002/jcb.26329 has nothing to deal with TCF7L2 , and merely by co-IP of β-catenin/TCF7L2 in chicken fibroblast(df1) may not be enough to conclude "Wnt5a-β-catenin- TCF7L2 positively regulates PGCs formation" . More direct evidence may be needed to support.

Thank you for your suggestion. The data in the manuscript of He et al., (2018) proved that Wnt signal mediated by WNT5A can promote the formation of PGCs. Our results show that Wnt5A participates in the formation of PGCs through β-catenin/TCF7L2 complex. Therefore, we come to the conclusion that: Wnt5a-β-catenin-TCF7L2 positively regulates PGCs formation。

In order to make the description in the manuscript more credible, we modified the description in the manuscript as follows(**Line114-112**):

Co-immunoprecipitation (Co-IP) of lysates of PGCs co-transfected with pcDNA3.1-Myc-*TCF7L2* and pcDNA3.1-*β-Catenin* revealed that TCF7L2 and β-catenin interact (Fig. 1E). These data indicate that Wnt5A mediates Wnt5A/β-catenin/TCF7L2 signaling in PGCs. We previously confirmed that Wnt5A signaling promotes PGCs formation (Fig. 1F), The results of QRT-PCR during the formation of PGCs in vivo and in vitro showed that the expression of *Wnt5A*, *β-Catenin* and *TCF7L2* in ESCs was far lower than that of PGCs (Fig. 1D), Combined with the results of Co- IP (Fig. 1E), we come to the conclusion that Wnt5a-β-catenin-TCF7L2 positively regulates PGCs formation.

Reviewer 1-12 Most of the experiments using overexresssion/ knockdown of specific gene in analyzing its effect on inducing PGCs the gain/loss of function assays are logically sound, but the co-ip experiment are conducted and mirn agga-let-7a-2-3p (mimic and inhibitor with Blimp1- 3'UTR-WT and Blimp1-3'UTR-Mut.) are conduct in chicken fibroblast(df-1), I think they should be done in induced PGCs or at least change their wording .

Thank you for your suggestion. In order to make data in the manuscript more convincing, we supplied the relevant experimental results in PGCs and iPGCs. Details are as follows:

In Fig.5: We carried out co-IP experiment in PGCs to prove the interaction between LSD1, TCF7L2 and β-catenin, and the results were consistent with the experimental results in DF-1 cells. The result in PGCs was supplied in Fig.5, and the result in DF-1 was supplied in S Fig6.

Figure 5 Competition of β-catenin and LSD1 in the formation of PGCs in combination with TCF7L2 regulates the expression of Lin28A . A. Effects of Wnt Signaling on the Regulation of Lin28A Promoter Activity After MLL2 and LSD1 Interference were detected by dual luciferase system. B,C. Results of Co-IP showed that LSD1 cannot bind to β-Catenin, but bind to TCF7L2 in PGCs. D. Results of Co-IP showed thatβ-Catenin competes with LSD1 binding to TCF7L2 in PGCs.

Supplementary Figure 6 Results of Co-IP showed the interaction of LSD1, TCF7L2 and β-Catenin in DF-1.

In Fig6: We conducted similar experiments in PGCs and iPGCs, the results of which are supplied in Figure 6.

Figure 6 [We have removed a figure which was provided to the reviewers in confidence]

© 2020. Published by The Company of Biologists under the terms of the Creative Commons Attribution License (https://creativecommons.org/licenses/by/4.0/).

Reviewer 2 Comments for the Author:

Reviewer 2-1 The present paper investigates the molecular mechanisms by which BMP4 and Wnt regulate PGCs differentiation in the chicken.Using both in vitro and in vivo approaches authors present a large number of results and the overall conclusions seem appropriate although the wealth of data does not always facilitate the deduction of a clear result and how it has been obtained. 8 figures and 8 supplementary figures, each containing at least 5 panels, cause confusion that should be cleared up. Some data have been previously published by the same authors or others. The new data should be presented more concisely. In other words results should be shortened and their interpretation should be moved to discussion section.

Thank you for your suggestion. We have modified the abstract, introdiction, results, methods section and Figures to make the contents of the manuscript easier to understand.(The revised part of the manuscript has been highlighted in red)

Reviewer 2-2 It is unclear how PGCs were isolated.It is unclear the source of ESC.

Thank you for your suggestion. In the method section of the manuscript, we supplied the separation method of PGCs and ESCs , we aslo explain the origin of ESCs. The details are as follows(**Line385- 401**): Cell isolation and culture

For ESCs：Chicken ESCs are derived from intracellular masses at the blastocyst stage. Fresh fertilized eggs were disinfected by 4% benzalkonium bromide and 75% alcohol. The blunt end of egg was broken by the tweezer and eggwhite was removed, then blastoderm cells at stage X were collected by the spoon method (Dai et al., 2007) in tissue culture dishes and rinsed With phosphate- buffered saline (PBS) to remove the yolks and vitelline membrane. After washing with PBS, ESCs were maintained in a 5% CO_2 humidified atmosphere at 37.0℃ with culture medium.

For PGCs：4.5d fertilized eggs were sterilized with 4% benzalkonium bromide and 75% alcohol to obtain chicken embryos to separate the genital bridge. After the genital bridge was cut and digested by trypsin for 3min, the cell suspension was obtained which filtered with 400 mesh filter cloth. After the cell suspension was centrifuged at 1600rpm/min for 6mim, the supernatant was discarded and the cells were collected. Cell precipitation was resuspended in PGCs medium.

KO-DMEM medium (Gibco, A3181501) contains 10%FBS, 2.5% chicken serum (Sigma, C5405), 100U/mL penicillin, 100U/mL streptolycin, 0.4μmol/L non-essential amino acid (Sigma, M7145), 2mmol/L glutamine (Sigma, G7513), 0.1mmol/Lß-mercaptoethanol (Sigma, M6250), 10ng/μL mLIF (Sigma, ESG1106), 10ng/μL bFGF (Sigma, GF446), 5ng/mLhSCF (Sigma, GF021) were used to culture ESCs and PGCs

Reviewer 2-3 Provide more details about how the in vivo experiments were performed.

Thank you for your suggestion. The detailed procedure of in vivo experiments is supplemented in the method section. The details are as follows(**Lin402-414**):

Vascular Injection of Chicken Embryo and Immunohistochemistry Experiment (IF)

Collect embryo from hatched eggs at 2.5 days (13-17HH), knock the round hole with a diameter not exceeding 0.5cm with tweezers at the blunt end. Expose the chicken embryo and use micro-pipettor inject the processed PGCs or the encased transfection vector into the embryonic blood vessel of the chicken embryo, then cross-sealed with medical tape to continue the incubation; collect the embryo at 4.5 days to prepare paraffin sections according to the previous procedure. Place the paraffin section in pH = 6.0, 0.01 M sodium citrate buffer solution, boiling in a microwave oven and take it to room temperature, repeat four times. 5% BSA diluted CVH primary antibody was incubate overnight at 4 ° C; wash the primary antibody with PBS three times and incubate secondary antibody for 1 h at room temperature, wash 3 times with PBS; use DAB-H_2O_2 for 10 min, Mayer staining 30 s, differentiation with hydrochloric acid and alcohol for 3 s, immersion in running water for 15 min; acetic acid treatment for 2 min, ethanol for 2 min, xylene for 5 min; cover the section within neutral gum, observe and take pictures by microscope(Nikon).

© 2020. Published by The Company of Biologists under the terms of the Creative Commons Attribution License (https://creativecommons.org/licenses/by/4.0/).

Reference:

Aige-Gil, V., and Simkiss, K. (1991). Sterilisation of avian embryos with busulphan. Research in veterinary science *50*, 139-144.

Chen, D., Yang, M., Xie, L., Lu, Z., Mo, L., Yang, W., Sun, J., Xu, H., Lu, K., Liao, Y., *et al.* (2020). GSK-3 signaling is involved in proliferation of chicken primordial germ cells. Theriogenology *141*, 62-67.

Lasko, P.F., and Ashburner, M. (1988). The product of the Drosophila gene vasa is very similar to eukaryotic initiation factor-4A. Nature *335*, 611-617.

Liu, Z., Cai, Y., Wang, Y., Nie, Y., Zhang, C., Xu, Y., Zhang, X., Lu, Y., Wang, Z., Poo, M., *et al.* (2018). Cloning of Macaque Monkeys by Somatic Cell Nuclear Transfer. Cell *172*, 881-887.e887.

Lu, Y., West, F.D., Jordan, B.J., Jordan, E.T., West, R.C., Yu, P., He, Y., Barrios, M.A., Zhu, Z., Petitte, J.N., *et al.* (2014). Induced pluripotency in chicken embryonic fibroblast results in a germ cell fate. Stem cells and development *23*, 1755-1764.

Nakamura, Y. (2017). Avian Biotechnology. Advances in experimental medicine and biology *1001*, 187-214.

Ono, T., and Machida, Y. (1999). Immunomagnetic purification of viable primordial germ cells of Japanese quail (Coturnix japonica). Comparative biochemistry and physiology Part A, Molecular & integrative physiology *122*, 255-259. Saitou, M., and Yamaji, M. (2012). Primordial germ cells in mice. Cold Spring Harbor perspectives in biology *4*.

Spudich, J.A., Gerhart, J., Mcknight, S.L., and Schekman, R. (1998). Annual review of cell and developmental biology. Annual Review of Cell and Developmental Biology *14*, V-V.

Tajima, A., Naito, M., Yasuda, Y., and Kuwana, T. (1998). Production of germ-line chimeras by transfer of cryopreserved gonadal primordial germ cells (gPGCs) in chicken. The Journal of experimental zoology *280*, 265-267.

Vick, L., Luke, G., and Simkiss, K. (1993). Germ-line chimaeras can produce both strains of fowl with high efficiency after partial sterilization. Journal of reproduction and fertility *98*, 637-641.

Wentworth, B.C., Tsai, H., Hallett, J.H., Gonzales, D.S., and Rajcic-Spasojevic, G. (1989). Manipulation of avian primordial germ cells and gonadal differentiation. Poult Sci *68*, 999-1010.

Yamaguchi, H., Xi, Y., and Fujihara, N. (2000). Inter embryonic (homo- or hetero-sexual) transfer of primordial germ cells between chicken embryos. Cytotechnology *33*, 101-108.

Yasuda, Y., Tajima, A., Fujimoto, T., and Kuwana, T. (1992). A method to obtain avian germ-line chimaeras using isolated primordial germ cells. Journal of reproduction and fertility *96*, 521-528.

Zhao, D.F., and Kuwana, T. (2003). Purification of avian circulating primordial germ cells by nycodenz density gradient centrifugation. Br Poult Sci *44*, 30-35.

Second decision letter

MS ID#: JOCES/2020/249375

MS TITLE: BMP4 activates the Wnt-Lin28A-Blimp1-Wnt pathway to promote primordial germ cells formation via altering H3K4me2

© 2020. Published by The Company of Biologists under the terms of the Creative Commons Attribution License (https://creativecommons.org/licenses/by/4.0/).

AUTHORS: Qisheng Zuo, Kai Jin, Yani Zhang, Guohong Chen, and Bichun Li
ARTICLE TYPE: Research Article

We have now reached a decision on the above manuscript.

To see the reviewers' reports and a copy of this decision letter, please go to: https://submit-jcs.biologists.org and click on the 'Manuscripts with Decisions' queue in the Author Area. (Corresponding author only has access to reviews.)

As you will see, the reviewers gave favourable reports but raised some critical points regarding the presentation of figures that will require amendments to your manuscript. I hope that you will be able to carry these out, because I would like to be able to accept your paper.

We are aware that you may be experiencing disruption to the normal running of your lab that makes experimental revisions challenging. If it would be helpful, we encourage you to contact us to discuss your revision in greater detail. Please send us a point-by-point response indicating where you are able to address concerns raised (either experimentally or by changes to the text) and where you will not be able to do so within the normal timeframe of a revision. We will then provide further guidance. Please also note that we are happy to extend revision timeframes as necessary.

Please ensure that you clearly highlight all changes made in the revised manuscript. Please avoid using 'Tracked changes' in Word files as these are lost in PDF conversion.

I should be grateful if you would also provide a point-by-point response detailing how you have dealt with the points raised by the reviewers in the 'Response to Reviewers' box. Please attend to all of the reviewers' comments. If you do not agree with any of their criticisms or suggestions please explain clearly why this is so.

Reviewer 1

Advance summary and potential significance to field

we appreciate the authors' major effort in responding to our comments and as a result, significantly improved the quality of data, proficiency of writing, with better logical flow throughout and increased clarity of the source datasets used for various analysis.

Comments for the author

Since the authors have prepared a graphic summary nicely summarizing their finding and their vision, it would be really a pity for not showing it in the paper at all. Although JoCeS does not usually accept graphic summary, I think the proposed Fig 9 can serve as the last main figure. Alternatively, in case 8 figures were the maximum allowed, the author can still put the graphic summary as supplementary figure. It does help the readers to understand the materials better.

The size of genital ridge on Fig 1f differ a lot, I assume that was the phenotype but just would like to double check that the magnification is consistent to all samples in each panel.

The authors state that whether chicken PGCs use the preformation or epigenesis model has not been clarified. I saw various papers stating that chicken belongs to the preformation category. I do not insist on this but the authors may consider discussing those other opinions.

I still do not 100% agree with the authors of using the logic flow of “limited number of chicken PGCs warrant the ESC to PGC induction protocol”. Again, I do not insist in this since different view points can be considered. In our hands chicken PGC can be amplified to a large quantity and can be used for both transgenesis and Crisper-cas9 editing.

© 2020. Published by The Company of Biologists under the terms of the Creative Commons Attribution License (https://creativecommons.org/licenses/by/4.0/).

Reviewer 2

Advance summary and potential significance to field

The present paper investigates the molecular mechanisms by which BMP4 and Wnt regulate PGCs differentiation in the chicken. Using both in vitro and in vivo approaches authors present a large number of results and the overall conclusions are appropriate, although the wealth of data does not always facilitate the deduction of a clear new result or a confirmation.
Data are not of broad interest to the cell biology community.

Comments for the author

In the revised version the authors readapted part of the text and some of the figures, without substantially making them easier to understand. Thus I still have the same concern as to the original manuscript. Although interesting, the paper is wordy and repetitive. For example in lines 69-91 the authors keep reiterating the concept that BMP4 and Wnt signaling play an important role in PGC formation. A schematic representation of the complex relationship among various factors involved in chicken PGC formation should be provided for the benefit of the reader.

Second revision

Author response to reviewers' comments

Dear Editor and Reviewers:

Thank you for your time and effort in dealing with our manuscript "BMP4 signaling activates the Wnt-Lin28A-Blimp1-Wnt positive feedback pathway to promote primordial germ cell formation via altering H3K4me2" (*JOCES/2020/249375*) in *Journal of Cell Sicence*. We are very pleased to see these comments, which are constructive and helpful to improve the quality of our study. Based on this, we carefully revised the manuscript and addressed all the comments raised by the reviewers point by point. We hope that this vision of our manuscript will meet the requirements for *Journal of Cell Sicence* publications.

We have addressed all the questions. The response was highlighted in Blue following each comment, which aslo highlighted with Blue in manuscript. The Red part in manuscript was traces of the last modification.

Reviewer 1 Comments for the Author:

Reviewer 1-1 Since the authors have prepared a graphic summary nicely summarizing their finding and their vision, it would be really a pity for not showing it in the paper at all. Although JoCeS does not usually accept graphic summary, I think the proposed Fig 9 can serve as the last main figure. Alternatively, in case 8 figures were the maximum allowed, the author can still put the graphic summary as supplementary figure. It does help the readers to understand the materials better.

Thank you for your suggestion. We have shown the Graphic Summary as supplementary figure 8D in the first paragraph of the discussion in manuscript, which will help the readers to understand the contents of the manuscript better.

Line292-297: The present study established a new regulatory model for PGCs formation. After activation of Wnt5A-β-catenin-TCF7L2 signaling, β-catenin competes with LSD1 to bind to TCF7L2 in the *Lin28A* promoter, leading to increased H3K4me2 levels and expression of *Lin28A*. Lin28A then activates expression of *Blimp1* by inhibiting the maturation of *Let7a*, thus regulating PGCs formation. Notably, *Blimp1* activates Wnt to initiate the Wnt-*Lin28*-*Blimp1*-Wnt positive feedback pathway(Fig. S8D).

© 2020. Published by The Company of Biologists under the terms of the Creative Commons Attribution License (https://creativecommons.org/licenses/by/4.0/).

Fig. S8D [We have removed a figure which was provided to the reviewers in confidence]

Reviewer 1-2 The size of genital ridge on Fig 1f differ a lot, I assume that was the phenotype but just would like to double check that the magnification is consistent to all samples in each panel.

Thank you for your suggestion. We examined our results carefully and confirmed that they were accurate.

Reviewer 1-3 The authors state that whether chicken PGCs use the preformation or epigenesis model has not been clarified. I saw various papers stating that chicken belongs to the preformation category. I do not insist on this but the authors may consider discussing those other opinions.

Thank you for your suggestion. Indeed, I have read a lot of relevant references, some of which clearly support that the Formation of PGCs belongs to 'preformation', for example:"DAZL Expression Explains Origin and Central Formation of Primordial examination in Chicken" (Jae Yong Han, 2015), I also support this view. However, in the course of our team's research, we found that PGCs formation in chickens has a very similar regulatory mechanism to that in mammals, so we were puzzled. Recently, in order to systematically determine the specific way of chicken PGCs origin, The single-cell RNA sequencing(10X) was performed to analyzed the composition of cell type in chicken embryos of different development periods, and we believe that results will be obtained soon.

Reviewer 1-4 I still do not 100% agree with the authors of using the logic flow of "limited number of chicken PGCs warrant the ESC to PGC induction protocol". Again, I do not insist in this since different view points can be considered. In our hands chicken PGC can be amplified to a large quantity and can be used for both transgenesis and Crisper-cas9 editing.

Thank you for your suggestion. Our research group has been conducting PGCs related researches for nearly 20 years. Although we have been constantly studying the formation mechanism and specific application of chicken PGCs, the problem of long-term PGCs culture has not been solved, unfortunately.

For the past 20 years, our team has been working on the development of PGCs. Although we continue to study the formation mechanism and specific application of PGCs in chickens, unfortunately, the problem of long-term PGCs culture has not been solved.

In the latest revision, we have focused on the molecular mechanism of PGCs, rather than on the application of PGCs. The detail are as follows:

Line34-35: We thus elucidated a positive feedback pathway involving Wnt-*Lin28*-*Blimp1*-Wnt, that ensures PGCs formation. In summary, our data provide a new insights in the development of PGCs in chickens.

Line50-54: Therefore, studying the molecular mechanism of chicken PGCs formation can ensure the application of PGCs. As we all know, the molecular mechanisms of PGCs formation in mammals are well studied, However, such research in PGCs chickens is not optimistic. Meanwhile, because of species differences, it is not clear whether the mechanisms of PGCs in mammalian can be applied to chickens.

Line90-92: Our results lay the foundation for the systematic analysis of the molecular mechanism of chicken PGCs formation, and also provide a new perspective to elucidate the origin of chicken PGCs.

Reviewer 2 Comments for the Author:

Reviewer 2-1 In the revised version the authors readapted part of the text and some of the figures, without substantially making them easier to understand. Thus I still have the same concern as to the original manuscript. Although interesting, the paper is wordy and repetitive. For example in lines 69-91 the authors keep reiterating the concept that BMP4 and Wnt signaling play an important

© 2020. Published by The Company of Biologists under the terms of the Creative Commons Attribution License (https://creativecommons.org/licenses/by/4.0/).

role in PGC formation. A schematic representation of the complex relationship among various factors involved in chicken PGC formation should be provided for the benefit of the reader.

Thank you for your suggestion. In order to make the content of the manuscript more concise and easy to understand, we modified the description of the manuscript. All the changes are marked in blue. For example:

Line73-80: However, other studies(Aramaki et al., 2013; Bialecka et al., 2012; Cervantes et al., 2009) confirmed that Wnt signaling also plays an important role in PGCs formation in mice. BMP4 and Wnt signaling interact during PGCs formation by activating *Blimp1* via the mesoderm-specific transcription factor T (Aramaki et al., 2013; Chassot et al., 2017; Yamaguchi et al., 1999). Specifically, In mouse epidermis, activation of BMP4 signaling and suppression of Wnt signaling leads to partial or complete loss of marker gene (such as *Blimp1*) expression in early PGCs (Ohinata et al., 2009; Saitou et al., 2005), whereas activation of Wnt signaling enhances the epidermal response to BMP4 signaling.

Line96-100: In order to analyze the molecular mechanism regulating chicken PGCs formation, The DEGs (Differentially expressed genes) of ESCs and PGCs were obtained by RNA-seq (Fig.1A,B). GO(Gene Ontology) analysis showed that 2516 DEGs were enriched in development-related terms (Fig. S1A,B and Table S1), 20 genes of which were significantly enriched in the Wnt signaling pathway (P=0.0023)(Fig. 1C, and Table S2). The result of qRT-PCR also comfirmed that the key molecule of Wnt signaling were significantly activated in the process of chicken PGCs formation (Fig. S1C,D).
We salo supplied a Graphic Summary as supplementary figure 8D in the first paragraph of the discussion in manuscript, which will help the readers to understand the contents of the manuscript better.

Line292-297: The present study established a new regulatory model for PGCs formation. After activation of Wnt5A-β-catenin-TCF7L2 signaling, β-catenin competes with LSD1 to bind to TCF7L2 in the *Lin28A* promoter, leading to increased H3K4me2 levels and expression of *Lin28A*. Lin28A then activates expression of *Blimp1* by inhibiting the maturation of *Let7a*, thus regulating PGCs formation. Notably, *Blimp1* activates Wnt to initiate the Wnt-*Lin28*-*Blimp1*-Wnt positive feedback pathway(Fig. S8D).

Third decision letter

MS ID#: JOCES/2020/249375

MS TITLE: BMP4 activates the Wnt-Lin28A-Blimp1-Wnt pathway to promote primordial germ cells formation via altering H3K4me2

AUTHORS: Qisheng Zuo, Kai Jin, Yani Zhang, Guohong Chen, and Bichun Li
ARTICLE TYPE: Research Article

I am happy to tell you that your manuscript has been accepted for publication in Journal of Cell Science, pending standard ethics checks.

© 2020. Published by The Company of Biologists under the terms of the Creative Commons Attribution License (https://creativecommons.org/licenses/by/4.0/).
